# Assistance systems for patient positioning in radiotherapy practice

**DOI:** 10.1080/20476965.2024.2395567

**Published:** 2024-10-28

**Authors:** Ralf Müller-Polyzou, Melanie Reuter-Oppermann, Jasmin Feger, Nicolas Meier, Anthimos Georgiadis

**Affiliations:** a Faculty of Management and Technology, Leuphana University, Lüneburg, Germany; b Faculty of Health, Medicine and Life Sciences, Care and Public Health Research Institute (CAPHRI), Maastricht University, Maastricht, The Netherlands

**Keywords:** Assistance system, patient positioning, radiotherapy, system design

## Abstract

Effective radiotherapy for cancer treatment requires precise and reproducible positioning of patients at linear accelerators. Assistance systems in digitally networked radiotherapy can help involved specialists perform these tasks more efficiently and accurately. This paper analyses patient positioning systems and develops new knowledge by applying the Design Science Research methodology. A systematic literature review ensures the rigour of the research. Furthermore, this article presents the results of an online survey on assistance systems for patient positioning, the derived design requirements and an artefact in the form of a conceptual model of a patient positioning system. Both the systematic literature review and the online survey serve as empirical evidence for the conceptual model. This paper thereby contributes to broadening the academic knowledge on patient positioning in radiotherapy and provides guidance to system designers.

## 1. Introduction

The world’s population is increasing and is expected to reach 9.198 million in 2040, with solid growth in the over-60 age group (Nations, 2021). Considering the demographic change and increasing cancer incidence rates, the number of new cancer cases is projected to reach 30 to 37 million per year in 2040 (Ferlay et al., 2019; The International Agency for Research on Cancer, 2024). The developments underline the need for transformation to secure effective and efficient healthcare (World Health Organization, & International Atomic Energy Agency Eds., 2020). The digital transformation in this context improves the quality of care for patients, reduces costs and enables individualised treatments (Gastaldi et al., 2012; Murphy, 2018). This is especially true for the personalised radiotherapy (RT) that 52 percent of cancer patients receive at least once as part of their cancer treatment (Atun et al., 2015). In radiotherapy, linear accelerators (linacs) are used to harm or destroy tumours with high doses of radiation. Modern conformal radiotherapy delivers the radiation dose to the tumour from different angles to maximise the dose while sparing healthy tissue (Schlegel et al., 2018). Overall, patients benefit from painless and precise treatment with fewer side effects compared to chemotherapy (Wooster Community Hospital, 2021). Radiotherapy follows strict processes and treatment regimes in which the radiation dose is applied to the tumour in a defined number of fractions. Patients must be precisely positioned at the linac for every radiotherapy fraction. The patient’s position during 3D imaging for treatment planning is the reference position. The reproducible positioning of patients is of utmost importance for efficient and safe radiotherapy (Rosenblatt & Zubizarreta, 2017). The patient positioning process, including the involved systems and specialised staff, was modelled in a previous work of our research group, resulting in a comprehensive radiotherapy process map (Müller-Polyzou et al., 2021).

Laser and Surface-Guided Radiation Therapy (SGRT) systems ensure the reference position is precisely restored at the linac. The position is subsequently verified by Image-Guided Radiation Therapy (IGRT) systems often integrated into linacs, such as Cone-Beam Computerised Tomography (CBCT) and Electronic Portal Imaging Devices (EPID) (*Guidelines for the Certification of Clinically Qualified Medical Physi- cists*, *2021*). Also, external X-ray-based systems have been used for many years and are now upgraded with camera technology (Brainlab, 2022). Possible deviations of the patient’s position are corrected by specially trained radiotherapy staff. Vendors continuously seek to optimise these tasks by integrating CBCT and EPID into linacs and treatment workflows (Varian, 2022). After the patient’s position has been successfully verified at the linac, the radiation dose is delivered to the patient. Modern SGRT systems offer additional features, such as intra-fraction motion detection and respiratory gating, increasing the safety and efficiency of therapy.

Assistance systems can help radiotherapy specialists by providing context-sensitive information, guidance through complex workflows and alert notifications. They can support efficient workflows and advanced decision-making, thus enabling more intensive personalised care. Assistance systems should close the gap between technology and the individual skills of the user to positively influence the treatment outcome (Maedche et al., 2016; Morana et al., 2017). They are, therefore, particularly suitable for the technology-intensive radiotherapy. This paper will analyse assistance systems for patient positioning in radiotherapy, increasing the knowledge base in the subject area. We will focus especially on the design of assistance systems and, consequently, on Design Science Research (DSR) conducted on radiotherapy assistance systems. DSR builds upon the knowledge from the problem and solution space (Vom Brocke et al., 2020). Therefore, we examine previous DSR studies from the application domain, considering the characteristic features of patient positioning tasks. These include that high positioning accuracy is required to secure safe and efficient treatment of patients. The actual positioning task is carried out in a group work scenario by specially trained Radiation Therapists (RTTs). In most cases, two RTTs interact with each other while using multiple positioning aids and operating technical devices in parallel. Furthermore, the work also requires empathy for patients. The therapies for children, the elderly and palliative patients are additionally complicated. We will also consider the technical developments in radiotherapy practice and experts’ and practitioners’ feedback from the field. Therefore, considering the typology of Hoang Thuan et al. (2019), we will answer the following two research questions: (1) To which extent do academic publications concerning the development of patient positioning systems employ application-oriented Design Science Research and (2) Which design requirements should apply for the development of new assistance systems for patient positioning.

To answer these questions, we conduct a systematic literature review (SLR) and a structured online survey among medical physicists in Germany. We derive system design requirements (DRs) based on the aggregated knowledge. Using the empirical evidence, we will also develop an artefact in the form of a conceptual assistance system for patient positioning supported by one dedicated use case. We then evaluate both with expert discussions and desktop research. The main contributions of this work towards the fight against cancer are four-fold: (1) DSR knowledge aggregation on patient positioning in radiotherapy, (2) presentation of empiric data supporting the development of patient positioning assistance systems, (3) formulation of design requirements for new assistance systems for patient positioning in radiotherapy, and (4) development of a conceptual assistance system for patient positioning in radiotherapy.

The paper is structured as follows: In Sections 2 and 3, we summarise the foundations and describe our research methodology. Then, Sections 4 and 5 present the structured literature review and online survey results. Section 6 outlines the design of the patient positioning artefact. We conclude in Section 7 with our knowledge contribution, a discussion of limitations and a summary and outlook. The appendix contains an overview of the SLR-identified literature, the online survey’s structured questionnaire, and additional data referenced in our paper. Our objective drives our work to contribute to developing more advanced solutions to improve cancer care.

## 2. Foundations

Analysing assistance systems for radiotherapy requires understanding the complex interactions in healthcare and related processes. Additionally, for DSR-based research, it is essential to build on a solid foundation in the problem and solution space of the application domain. The people, activity, context and technology (PACT) dimensions help us to structure the knowledge analysis and build-up (Benyon, 2020; Morana et al., 2017). The PACT dimensions reflect how people use technologies to perform activities in a specific context. Finally, a well-established framework shall support our design-oriented work (A. R. Hevner, 2007; A. Hevner & Chatterjee, 2010; A. R. Hevner et al., 2004).

### 2.1. Radiotherapy environment

**Figure 1.**
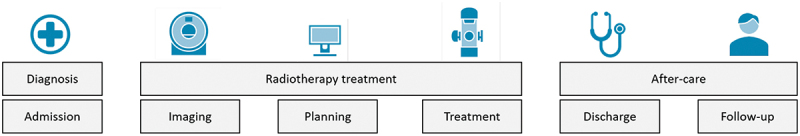
Process of radiotherapy treatment and underlying sub-processes (own figure based on (Müller-Polyzou et al., 2021)).

In previous work, we analysed the environment of modern radiotherapy with particular emphasis on digitalisation, technical systems and the staff involved (Müller-Polyzou et al., 2021). The results reflect the relationship between people, data and equipment in radiotherapy processes. The processes, including their sub-processes, were modelled, depicting a comprehensive process map of radiotherapy that represents the state of the art and can act as a denominator for individual workflows in global radiotherapy cancer treatment. At the highest process level, the radiotherapy process consists of three process steps: diagnosis, radiotherapy treatment and after-care, as shown in Figure 1. Diagnosis is, in turn, based on the patient admission sub-process, and treatment is based on the interdependent sub-processes of imaging, planning and treatment carried out in multiple treatment sessions. The process step after-care includes the sub-processes of discharge and follow-up. After the patient has been admitted, Computer Tomography (CT) and/or Magnetic Resonance Imaging (MRI) images are taken and used for treatment planning. The tissue to be irradiated and the radiation dose are defined in the treatment planning on computer-based planning systems. The treatment plan is then applied in several treatment sessions using the linac. After discharge, patients are monitored over a follow-up period of several years.

We also described and tested hypothetical assistance systems listed in Table 1 (Müller-Polyzou et al., 2021) that can help to improve workflows within radiotherapy departments. Systems two, three and four presented in Table 1 are related to patient positioning tasks.

**Table 1.** Hypothetical assistance systems according to (Müller-Polyzou et al., 2021).

No.	Section	Description
1	Digital anamnesis	Patients can enter their anamnesis data digitally on a mobile device.
2	Patient positioning	User assistance with photographic documentation of patient positioning and support of related tasks at the linac.
3	Immobilisation device assistance	Immobilisation documentation of patient at CT/MRI or Positron Emission Tomography (PET)-CT.
4	Patient validation	Patient validation at the linac with patient identifying technologies.
5	Imaging information system	User assistance with information about the type and location of tumours, displaying diagnostic images and providing information about the CT/MRI or PET-CT scan.
6	Therapy planning and scheduling	User assistance to coordinate, document and monitor irradiation planning.
7	Appointment planning	User assistance for appointment planning for medical physicists.
8	Risk management	User assistance that supports the monitoring, recording and archiving of relevant risk parameters.

**Figure 2.**
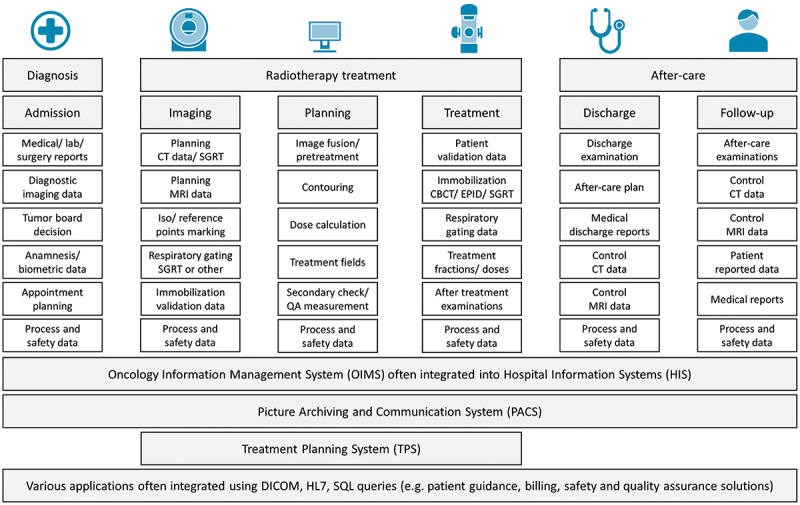
Process of radiotherapy treatment, corresponding data sources and underlying IT systems (own figure based on (Müller-Polyzou et al., 2021)).

The findings motivate our research and knowledge build-up on designing interactive user assistance systems embedded in the radiotherapy work and quality assurance processes to improve efficiency and reduce treatment risks. According to Maedche et al. (2016), advanced user assistance systems provide context-aware guidance or advice features. They need to be integrated into IT systems to access data. Digital radiotherapy is data intensive, and specialists must deal with an increasing amount of distributed data (Rosenblatt & Zubizarreta, 2017). Various authors have described and studied this situation regarding using artificial intelligence in radiotherapy (Field et al., 2021; Parkinson et al., 2021; Skripcak et al., 2014). Assistance systems with varying degrees of intelligence and interactivity can utilise the data to provide information to users with a view to efficient and safe radiotherapy. Figure 2 presents the radiotherapy process with the underlying data sources and IT systems. It demonstrates the wide range of data sources and information systems that assistance systems can utilise. The presentation was developed in intensive discourse with medical physicists utilising our previous research findings and existing literature (Schlegel et al., 2018; Müller-Polyzou et al., 2021).

### 2.2. Assistance systems

Assistance systems are one form of socio-technical information system that supports users in their task fulfilment. They are based on information technologies and include components developed with engineering and mathematical competencies (Benyon, 2020). The development of assistance systems requires a deep understanding of the subject area from the perspective of the involved business stakeholders (Clarke et al., 2020). Several classifications of user assistance systems have been developed for information systems. Morana et al. (2017) define user assistance in Healthcare Information Systems “[…] as a people-, activity- and context-dependent augmentation of task performance by bridging the gap between technology and people’s capabilities to influence task outcomes positively”. They utilise the level of interactivity and intelligence of assistance systems according to Maedche et al. (2016) and define three modes which are based on the level of user-system interactivity: (1) supportive mode, (2) cooperative mode, and (3) notifying mode. The definition of Morana et al. (2017) is used in our applied science work due to the high proportion of human-technology interaction in radiotherapy and its general applicability to healthcare information systems.

### 2.3. Patient positioning systems

**Figure 3.**
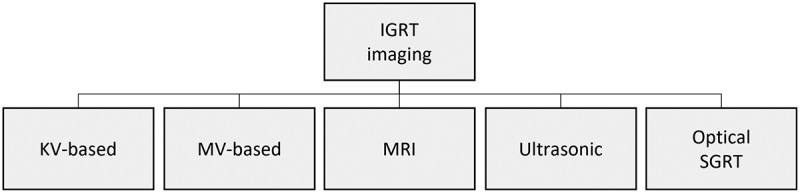
Overview on image guided radiotherapy Schlegel et al. (2018).

The reference for patient positioning in radiotherapy is defined during CT imaging for treatment planning. Additional MRI scans for specific tumour types are performed to add soft tissue information. At a later stage of treatment, the reference position of the patient is reproduced at the linac for every treatment session. As presented in Figure 3, IGRT solutions support reproducible patient positioning at the linac. Kilovoltage (KV) X-ray solutions, such as modern CBCT solutions, are nowadays integrated into linacs. They are accurate but apply additional radiation to patients. Megavoltage (MV) solutions are detectors integrated into the radiotherapy beam path. MRLinac accelerators combine MRI imaging with the linac in one medical device. Also, ultrasonic solutions can be used for IGRT. Both ultrasonic and MRLinac solutions work in real-time and provide soft tissue information. Optical SGRT solutions scan the patient’s body surface with camera technologies. They provide real-time 3D data without applying radiation dose (Hoisak et al., 2020; Schlegel et al., 2018).

Laser systems forming an independent coordinate system are the global standard for patient positioning. Laser systems project laser crosses onto the patient’s skin or immobilisation devices during CT/MRI imaging. After marking the patient, laser systems installed in the treatment room allow the patient to be accurately positioned at the linac (Hoisak & Pawlicki, 2018; Hoisak et al., 2020). Laser systems combined with KV portal imaging are used to verify the patient’s position. Camera-based SGRT systems are increasingly deployed and provide additional features such as intra-fraction motion control (Padilla et al., 2019). The systems also support special treatment techniques such as respiratory gating (Hoisak & Pawlicki, 2018). In this process, tumours are irradiated at specific time slots during a respiratory cycle, sparing organs at risk (Richter et al., 2009). To position the patient and to monitor potential movements, the patient’s body surface at the linac is either scanned before treatment or the patient’s surface created during the planning CT (Hombrink & Promberger, 2020) is used as reference.

Various commercial SGRT systems and combinations thereof are available, with an expert community supporting the dissemination of systems in the global radiotherapy market (Brainlab, 2022; C-RAD, 2022; Varian, 2021; Vision RT, 2021b, 2021c). The American Association of Physicists in Medicine (AAPM) reacted to the increased deployment of SGRT systems with the definition of guidelines for Surface Image Guided Radiotherapy in the Task Group TG302 (American Association of Physicists in Medicine, 2021). The gold standard for patient positioning in terms of accuracy and final treatment decisions are CBCT systems. The drawback of CBCT is the exposure of patients to additional radiation. Guo et al. (2020) provide a concise overview and comparison of patient positioning systems. Batista et al. (2020) outline the potential of SGRT to cause a paradigm shift in radiotherapy. The data provided by such systems shed light on the benefits of further SGRT development as a central tool promoting patients’ safety, treatment quality, motion management, risk surveillance and management. The authors argue that SGRT is more than a positioning tool. Their statement supports our previous analysis of assistance systems in radiotherapy and further encourages our design work.

## 3. Research methodology

**Figure 4.**
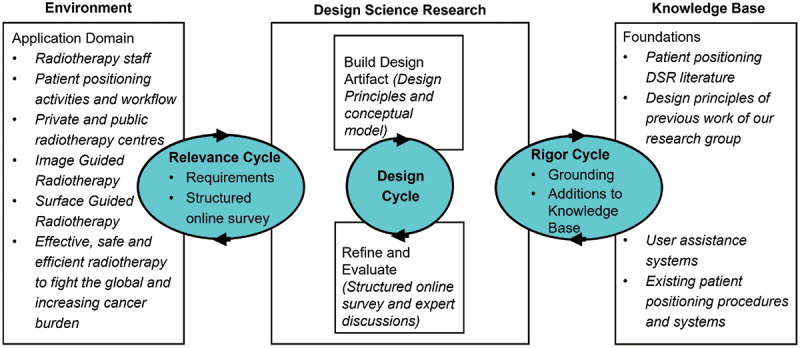
DSR concept according to A. Hevner and Chatterjee (2010).

**Figure 5.**
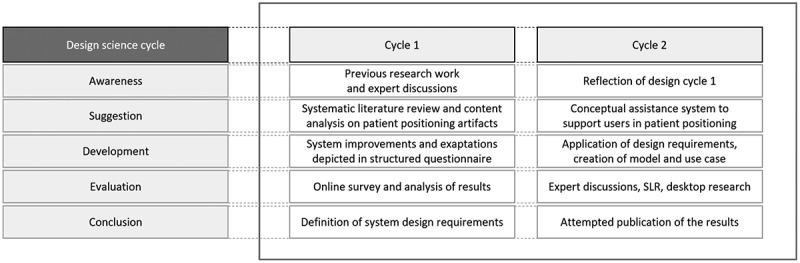
DSR cycles according to Vaishnavi and Kuechler (2015).

’’Rather than producing general theoretical knowledge, design scientists produce and apply knowledge of tasks or situations in order to create effective artefacts’’ (March & Smith, 1995). Against this background, we chose Design Science Research (DSR) as the research framework for our work. DSR supports the development of knowledge and artefacts for real-life problems while building on kernel theories. Applied kernel theories are interdisciplinary and include computer and information system science. DSR work consists of the relevance, rigour and design cycles shown in Figure 4 (Gregor & Hevner, 2013; A. R. Hevner, 2007; A. Hevner & Chatterjee, 2010). The relevance cycle bridges the radiotherapy application area with design activities, while the rigour cycle connects the design science activities with the overall knowledge base. The central design cycle builds and evaluates the artefacts as described by A. R. Hevner (2007). Figure 5 presents our work in the framework of Vaishnavi and Kuechler (2015).

The development of medical systems requires the consideration of particular aspects. Depending on the intended use of the system, additional requirements have to be fulfilled for risk management, usability and system safety (*IEC 62,366–1:2015: Medical devices – Part 1: Application of usability engineering to medical devices*, *2015*; 2019). DSR explicitly considers the involved actors’ perspectives when developing solutions. This is particularly important for understanding the social dimension of assistance systems (van Aken et al., 2016). The knowledge of the radiotherapy environment, IT systems, assistance needs, and patient positioning, combined with the DSR framework, provide a sound foundation for our research. In this context, DSR research for radiotherapy differs from other applications in healthcare due to the high utilisation of technology and specialised staff, considering high patient and staff safety requirements. Therefore, in contrast to many healthcare IS publications, a multi-perspective research approach was implemented to address the identified challenges of the subject field following the insights of Clarke et al. (2020); Ostern et al. (2021); Stephanidis et al. (2019).

Research methodologies support our design science work to produce new knowledge, deepen our understanding of the subject area and support the creation of interactive assistance systems. We apply qualitative and quantitative research methods for the different phases of our project.

### 3.1. Systematic literature review

A systematic literature review is efficient for analysing the existing knowledge base. The results provide a sound basis for the development of the online survey and the concluding formulation of knowledge additions. The review follows a systematic, transparent and reproducible process (Cooper, 1988; Tranfield et al., 2003; Webster & Watson, 2002). The search strategy applied according to the approach of Dresch et al. (2015) was broad in scope and is documented in Table 2. The search, which was conducted in Scopus and ScienceDirect included, among others, the journals of the American Society for Radiation Oncology (ASTRO), the European Society for Therapeutic Radiology and Oncology (ESTRO) and the journals of the Association for Information Systems (AIS). A deliberately broad search was conducted in phase one to avoid excluding relevant literature by choosing too narrow keywords. The selection took place in the screening process on the basis of the abstracts. The search terms, as presented in Table 3, were defined based on discussions with medical physicists and the index of a textbook of radiation physics (Schlegel et al., 2018). They were classified into the categories: (1) information systems, (2) radiotherapy, and (3) radiotherapy process steps, with additional synonyms being added to the search string. Searches were performed by title, abstract, and keywords. The search was conducted to identify information system publications related to external beam radiotherapy, its procedures and applications. Only journal articles, conference papers, book chapters, and editorials published in English up to September 11 2020 were considered. The literature management software Citavi was used for screening and categorising literature (CITAVI, 2021). The articles passing the inclusion criteria were first sorted according to radiotherapy processes as presented in Table 4 and subsequently according to DSR artefacts defined by A. Hevner and Chatterjee (2010) and shown in Table 5 in order to increase our understanding of DSR application in the field.

**Table 2.** Systematic literature review protocol of phase one.

Section	Description
Conceptual frame	Cancer as a leading cause of death requires efficient and effective therapies. Information systems in the form of assistance systems support users in their task fulfilment.
Context	Patient positioning in radiotherapy treatment
Period	Unlimited time period
Language	English
Strategy	Aggregating search
Focus	Title, abstract, keywords
Search string	(information systems and synonyms) AND (radiotherapy and synonyms) AND radiotherapy process steps
Inclusion criteria	External beam radiotherapy; IS artefacts; empiric data for information system development
Exclusion criteria	No relation to radiotherapy; no information system artefacts; clinical studies
Sources	Scopus and ScienceDirect

**Table 3.** Search terms of the systematic literature review.

Database	Search terms connected with Boolean AND command
Scopus	**user assistance** OR: expert system, decision support system, decision making system, decision aid, knowledge based system, information system, support system
	**radiotherapy** OR: teletherapy, radiation therapy, external beam radiotherapy, radiation oncology
	**admission** OR: informed consent, patient positioning, work-flow, scheduling, planning, validation, tumour board, Surface Guided Radiation Therapy, simulation, laser, quality assurance, risk management, model, tool, interactive, intelligent, prediction, application, device, platform, management, patient position, tattoo, immobilisation, sgrt, sigrt IGRT, surface-guided, surface guided, surface-based, surface-scanning, surface tracking, image-guided, image guided, image guidance, motion tracking, motion management, optical surface, guided radiotherapy
ScienceDirect	**decision aid** OR: knowledge based system, information system, support system
	**user assistance** OR: expert system, decision support, decision making
	**radiotherapy** OR: teletherapy, radiation therapy, external beam radiotherapy, radiation oncology

**Table 4.** Radiotherapy process steps applied for literature categorisation.

Process	Description
Diagnosis	Procedures and solutions related to specific diagnosis tasks for radiotherapy treatment.
Therapy decision	Procedures, solutions and tasks for patient-specific therapy decision-making.
Admission	Procedures and tasks for patient admission in radiotherapy centres.
Imaging	Imaging tasks using CT, MRI, PET-CT, or other devices.
Planning	radiotherapy treatment planning with expert software.
Therapy	radiotherapy treatment with linacs applied in a course of defined dose fractions.
After-care	Patient-specific tasks following the radiotherapy treatment course.

**Table 5.** DSR artefacts used for literature sorting according to (March & Smith, 1995).

Artefact	Description
Constructs	Formalised or informal terms used to describe problems and solutions within a domain.
Models	Set of propositions or statements expressing relationship among constructs. A description or representation of how things are.
Methods	Set of steps used to perform a task e.g algorithms or guidelines. Methods are based on underlying constructs and a representation model.
Instantiation	Instantiations implement constructs, models and methods. They demonstrate the feasibility of models and methods in prototype systems.

**Figure 6.**
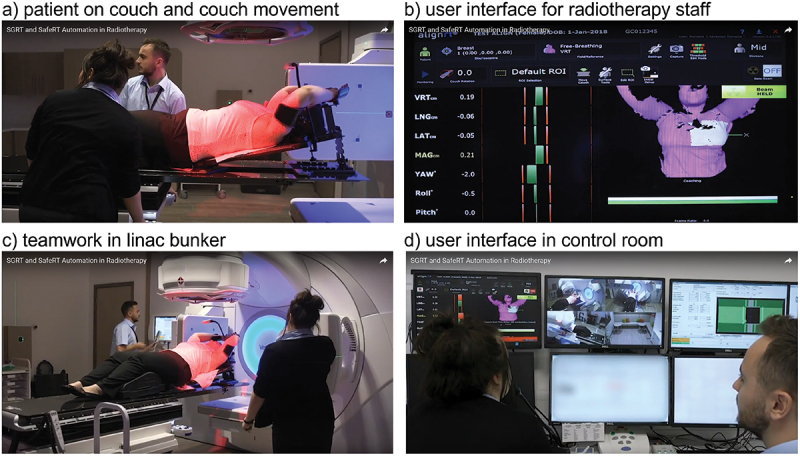
Examples of SGRT system usage reflecting the PACT dimensions (SGRT Community, 2020).

In phase two of the SLR, we focused on SGRT due to its potential for further innovation. To reflect the central role of SGRT, we analysed the process areas *imaging* and *therapy* with a focus on SGRT-related patient positioning instantiations (Hsieh & Shannon, 2005). The search was broadened with a backward and forward search before assessing eligibility. After that, a qualitative content analysis was performed on the remaining papers. The PACT concept for interactive socio-technical systems was applied for coding (Benyon, 2020). The people dimension in the PACT concept reflects physical, psychological, mental and social differences impacting the usage of systems. Activities are distinguished by their cooperation, complexity, temporal and safety-critical characteristics, and demands on human-system interaction. The PACT concept also reflects the context of the socio-technical system usage. The context is analysed in social, organisational and physical dimensions. The technology analysis focuses on the input and output modalities and communication and content factors (Anderson et al., 2011). Figure 6 shows selected works steps using an SGRT patient positioning system to visualise the corresponding PACT dimensions. In this context, Figure 6a) indicates the patient positioning procedure with two therapists moving the patient couch. Figure 6b) presents a typical user interface for the radiotherapy staff showing the patient’s body surface and reference positions. The physical work environment is visible in Figure 6c) with the two-person team working in the radiotherapy bunker and Figure 6d) at the control panel outside the radiotherapy bunker.

Based on the PACT dimensions, a mixed form of inductive-deductive coding was used according to Gläser, J., & Laudel, G. (2012). During the analysis, the codes were gradually revised and optimised (Mayring, 2000). The qualitative content analysis was performed with the software ATLAS.ti (ATLAS.ti, 2021).

### 3.2. Systematic online survey

To assess the level of user assistance for patient positioning in German radiotherapy practice, an online survey was designed and implemented with the tool Limesurvey (Limesurvey, 2021). The questionnaire was reviewed and tested by three radiotherapy experts and one medical physicist working at a private radiotherapy centre. The survey was optimised for high acceptance with a particular focus on the welcome message, the time needed to complete the questionnaire and the formulation of the questions (Schnell et al., 2013). In the beginning, the participants were informed about the scientific background of the survey and the time available to complete the questionnaire. In addition, they were assured of the anonymity of their responses. Most questions were formulated as closed-ended or semi-open questions to achieve a high degree of objectivity. A 5-point Likert scale was used to specify the level of agreement or disagreement with specific statements. The answers were either non-mandatory, or an”I don’t know”option was provided. Before submitting the results, the participants could provide additional information or comments in a free-text field. The questionnaire structure is shown in Table 6, while the complete questionnaire is available in the Appendix to assure reproducibility according to Cram et al. (2020).

**Table 6.** Structure of the questionnaire.

Section	Description
Opening	Welcome and personal introduction; goals; picture of researcher; survey duration; donation announcement
General information	Type of radiotherapy centre; number of linacs and staff; number of patients treated per year
Positioning at linac	Patient positioning systems and usage today and in two years; positioning and movement detection accuracy needs; required time for positioning tasks; improvement ideas
Positioning with SGRT	Information needs for tasks; barriers for system introduction; future SGRT features; patient-related questions
PACT	Importance of people experience; system features; system impact; integration aspects; human-machine interfaces (HMI)
Closing	Thank you message and contact details

## 4. Results of the systematic literature review

**Figure 7.**
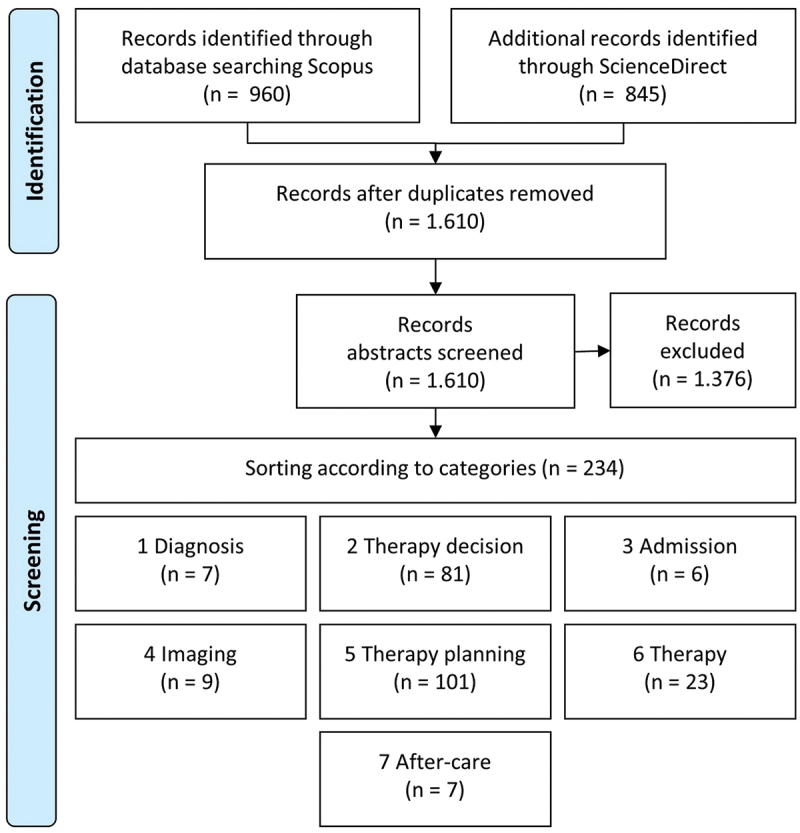
PRISMA for screening of DSR instantiations in radiotherapy process steps.

In the first phase of the SLR, 1,805 articles were identified and classified according to the defined categories. Duplicates were removed, and articles that did not meet the inclusion criteria were sorted out. The results are presented in the *Preferred Reporting Items for Systematic Reviews and Meta-Analyses* (PRISMA) according to Moher et al. (2009) and Liberati et al. (2009) shown in Figure 7.

In summary, the SLR of phase one reveals 234 publications related to information systems. Table 7 shows the number of publications according to the radiotherapy process steps and DSR artefacts. Most of the publications (77.7 %) are related to the process steps *therapy decision making* (81 publications) and *planning* (101 publications). Only 32 publications (13.7 %) address the process steps *imaging* (9 publications) and *therapy* (23 publications). Excluding the review articles, artefacts are described in 213 publications. Constructs (69.5 %) are most frequently described, followed by instantiations (18.3 %) and methods (10.3 %). Only a few models (1.9 %) are described in the identified literature. Most instantiations have been developed for the process step *therapy decision making* (38.5 %), while *diagnosis*, *admission* and *after-care* are underrepresented, indicating a need for further research.

**Table 7.** Cooccurrence of radiotherapy process steps and it artefacts.

Process	Constr.	Models	Methods	Instant.	Review	Total
Diagnosis	2	0	1	0	4	7
Therapy decision making	44	3	11	15	8	81
Admission	6	0	0	0	0	6
Imaging	3	0	2	2	2	9
Therapy planning	81	1	6	8	5	101
Therapy	8	0	1	12	2	23
After-care	4	0	1	2	0	7
Total	148	4	22	39	21	234

**Figure 8.**
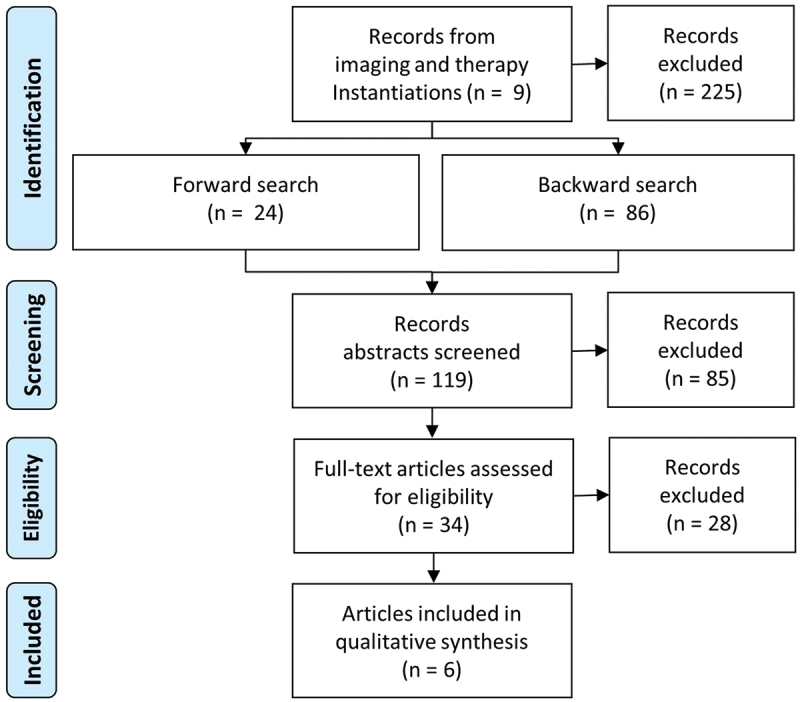
PRISMA presentation of search process for patient positioning screening.

In the *imaging* and *therapy* process steps, 14 articles were identified describing information system instantiations. Of those, nine articles dealt with patient positioning. A forward and backward search focusing on SGRT was performed, adding six articles eligible for full-text assessment, as shown in Figure 8 and listed in Table 8.

**Table 8.** Identified DSR literature for patient positioning instantiations.

Authors	Title	Focus
Calow et al. (2002)	Photogrammetric measurement of patients in radiotherapy	Camera system
Posada et al. (2007)	Towards a noninvasive intracranial tumour irradiation using 3D optical imaging and multimodal data registration	Camera system
Posada-Gomez et al. (2007), Gilles et al. (2016)	An extrinsic calibration method for 3D range surface sensors: an application in radiotherapy patient positioning	3D sensors with laser system
	Patient positioning in radiotherapy based on surface imaging using time of flight cameras	ToF camera
Fuse et al. (2017)	An infrared interactive patient position guidance and acquisition control system for use during radiotherapy treatment	IR camera
Cosentino et al. (2017)	RAD-AR: An augmented reality tool for radiotherapy implemented on consumer electronics devices	AR glasses

**Figure 9.**
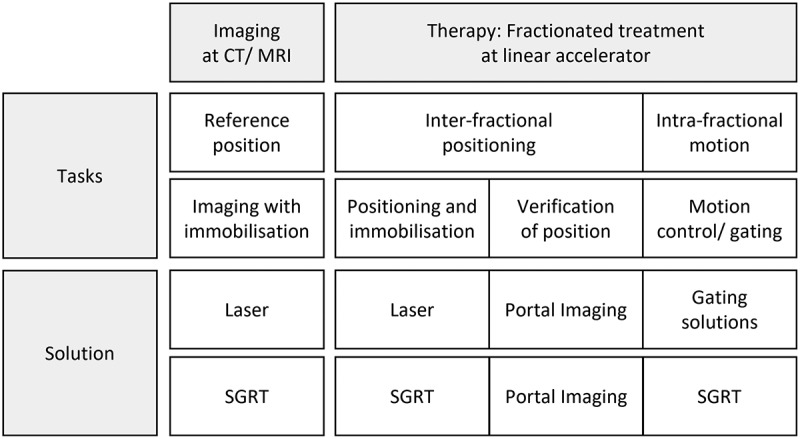
Tasks and solutions for patient positioning in imaging and therapy process steps (own figure).

Figure 9 illustrates the relationship between the process steps of patient positioning, the work tasks and the corresponding solutions. The reference position for *imaging* is established using immobilisation devices, while inter-fraction positioning and intra-fraction motion control are performed for *therapy*. In principle, patient positioning can be performed with laser and SGRT systems. Compared to laser systems, SGRT systems are sensory-based and support features such as intra-fraction motion detection and respiratory gating. Furthermore, the camera technology and its integration into the clinical network enable the development of new innovative features. The following content analysis was subsequently performed with the identified SGRT articles concerning the improvement possibilities of SGRT systems. Additionally, the increasing availability of SGRT systems in clinical routine provides an interesting basis for further enhancements and design evaluations.

**Figure 10.**
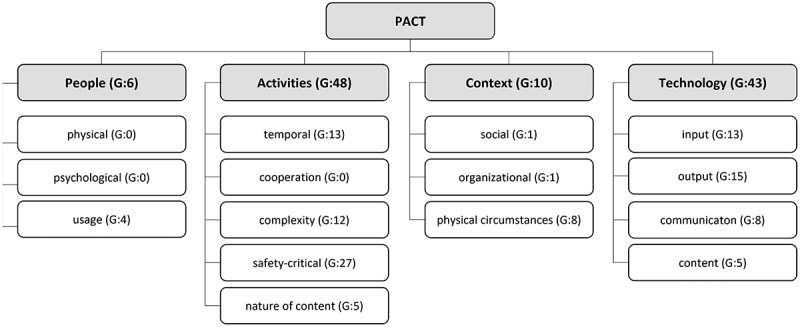
Code groundedness (G) of first and second-level code categories (own figure).

The content analysis of the identified literature was performed using an inductive-deductive coding approach. Code networks were developed to present the conceptual relationships between codes. For this purpose, the codes were symmetrically, asymmetrically or transitively linked. The measures for assessing the importance of codes are the frequency of code usage and the density of the connections within the code network. The PACT dimensions were used as starting points in the code network, while the first level of codes comprises the subcategories of the PACT dimensions as described in Table 9 and shown in Figure 10. The inductively elaborated process forms additional codes. The results were afterwards used to structure and formulate the questionnaire for the online survey.

**Table 9.** PACT dimensions and first-level codes.

Dimension	Codes	Description
People	Physical	Differences in appearance, cognitive abilities and sensory perceptions.
	Psychological	Perception shaped by culture and language.
	Usage	Differences in use by various user groups.
Activity	Temporal	Describes the frequency of activities.
	Cooperation	Activities are performed alone or with others.
	Complexity	Steps needed to complete tasks as well as complexity in communication and coordination.
	Safety critical	Aspects that may result in injury or accident.
	Nature of content	Requirements arising from the data generated by the activity.
Context	Social	Influences of the environment on activity.
	Organisational	Activity location and influence on the organisation.
	Physical	Place of activity and resulting external influences.
Technology	Input	Technology used to enter commands or data.
	Output	Technology used to output data to users.
	Communication	Human-machine communication in terms of speed and bandwidth.
	Content	Form in which the data is available in the system.

The publications describe various technical solutions for acquiring body surface data. Among the solutions are: (1) photogrammetry-based camera systems with structured or scattered light projection, (2) ToF camera systems measuring the distance from the camera to the body surface, (3) IR markers that are placed on the patient’s body and recognised by cameras, and (4) an augmented reality solution for body surface measurement and information presentation. The DSR publications seem to follow the development of technology.

The analysis of the PACT dimensions presented in Table 9 shows that the activities (44.9 %) and the technology dimension (40.2 %) are firmly grounded (G) in the publications. The people (5.6 %) and context (9.3 %) dimensions are only slightly addressed in the identified literature, which is notable for socio-technical information systems.

The analysis of the codes in the sub-dimensions shows that the accuracy and safety aspects of the systems are critical. In addition, time aspects are often mentioned, especially concerning the reduction of the patient positioning time, which is a clear denominator for efficiency. It also emerges that low system complexity and intuitive system usability is desirable. Sensors, cameras, tablets and phantoms were mentioned for data acquisition. For the visualisation of information, tablets, AR glasses, lasers and projectors were mentioned in the publications. In summary, the articles focus on effectiveness, efficiency, usability and safety. User requirements for applicability within the overall context of radiotherapy are not considered. The SLR results increase our design knowledge in the problem and solution space according to Vom Brocke et al. (2020). They also inform the empirical work and have been incorporated into the questionnaire design. In addition to the justificatory knowledge, informal knowledge from the application area and experience from practitioners is used following Gregor and Hevner (2013).

## 5. Results of the structured online survey

### 5.1. Sample group and implementation

The structured online survey was designed for medical physicists as practitioners from the application field. Medical physicists are specially trained and responsible for the technical systems and safety in radiotherapy, including patient positioning systems (*BERUFENET Medizinphysiker/in*, Berufenet medizinphysiker/in, 2022; Guidelines for the Certification of Clinically Qualified Medical Physicists, 2021; *MPE in der Strahlen- und Brachytherapie*; 2022; Rosenblatt & Zubizarreta, 2017). The safety-relevant tasks of medical physicists require an intensive exchange with the operative staff and the management of the radiotherapy department. They are, therefore, well-informed on relevant issues, even across different stakeholder viewpoints. The survey was implemented in German and was accessible from 2 to November 15 2020. The participants were selected from the members of the German Association for Radiation Oncology (DEGRO, 2021). The association represents radiotherapy departments and centres in Germany. At least three radiotherapy centres per federal state were randomly selected to ensure a nationwide distribution. Overall, 90 radiotherapy centres were contacted by telephone, of which 12 centres refused to participate due to time concerns or limited resources. Therefore, the survey was sent out in a personalised email to 78 radiotherapy centres, of which 68 participated and 45 fully completed the questionnaire. Only the fully completed questionnaires were evaluated. This means that approximately 15 percent of radiotherapy centres in Germany participated in the survey. Tables A1 and A2 in the appendix present sample group details. In summary, the sample group reflects the German radiotherapy market. However, it is a non-representative and random selection providing expert insights into the specific field of radiotherapy, particularly the critical task of patient positioning.

### 5.2. Empirical data – general

The patient positioning tasks at the linac were investigated as a first step. To this end, the participants were asked which systems they currently use. We also asked about the usage frequency of those systems concerning the total number of patients treated per year. Subsequently, the participants were asked what changes they expected in the availability of patient positioning systems within two years. The results are presented in the appendix in Table A3. All 45 radiotherapy centres use IGRT and foresee that they will continue to use IGRT within the two-year time frame. IGRT solutions are an integral part of the patient positioning workflow. At the time of the study, 41 of the 45 radiotherapy centres (91.1 %) used laser systems. This number is expected to decrease by 15.5 percentage points within two years. Thirty-four participants (75.6 %) state that they will use laser systems by the end of the year 2022. SGRT systems were being used by 16 radiotherapy centres (35.6 %). An increase of 35.5 percentage points is expected within the next two years, with 32 participants (71.1 %) believing or knowing they will use SGRT systems by the end of 2022. Additionally, the experts mentioned third-party X-ray-based systems (3 participants) and the linac’s light-field (3 participants) as positioning aids being used today. These systems will continue to be used in the timeframe mentioned above. However, two participants stated that they would then be in the process of purchasing gating and SGRT solutions. The participants were also asked about the frequency of usage of the various positioning systems. The answer was not mandatory and was designed as a free text field to allow additional information and comments. Thus, the usage frequency of the systems was calculated manually as the average of the provided answers. The frequency of answers and standard deviation are presented for transparency reasons.

The usage of IGRT systems that are utilised for 81.5 % of the patients is expected to decrease by 3.3 percentage points by 2022. Laser systems that are used for 87.2 % of the patients are expected to experience a decrease of 9.8 percentage points in their usage. However, the frequency of usage of SGRT systems is foreseen to increase by 12.1 percentage points in the time frame, reaching 66.5 %. Laser systems are available in almost all RT centres. They are reliable, technically independent and easy to use. Additionally, they support quality assurance tasks such as positioning phantoms for dose measurements. This fact could explain the still high expected use of laser systems.

Accuracy and time are essential for safety and efficiency in radiotherapy. When asked about the minimum accuracy required for inter-fraction patient positioning, participants (*n* = 45) named an average accuracy of 2.8 mm with a standard deviation (SD) of 1.6 mm. 84.4 % of the participants expect a positioning accuracy of 3.0 mm. After positioning, the patient can still move by reflexes such as swallowing, sneezing or coughing. When asked about the required detection of intra-fraction movement, participants (*n* = 45) stated that movement changes of 2.4 mm on average (SD = 3.2 mm) should be detected. These results show a high standard deviation, possibly because of the different treatment regimes. To be future-proof, patient positioning systems should support a higher accuracy in support of stereotactic irradiation and hypofractionation with higher doses, fewer fractions, and lower contouring margins. When asked about the time required for patient positioning, participants (*n* = 42) reported 5.3 minutes on average. This value is consistent with the authors’ experience. German radiotherapy centres often allocate slots of 10 minutes per patient for recurring fractions in the linac room. However, times required for patient positioning vary depending on the system used, as shown in Table A5 in the appendix. Positioning with laser systems requires, on average, 4.3 minutes. The time needed using both laser and SGRT systems increases by 41.9 % to 6.1 minutes. The time required using only SGRT systems climbs to 9.0 minutes. This fact seems plausible, but the result is only based on the responses of three participants.

Concluding the section about *patient positioning at the linac*, the participants were asked about measures that could simplify the patient positioning task. Participants called for better integration of SGRT systems with OIMSs and linacs and additional control features related to imaging and salient points of the body. Finally, it was pointed out that SGRT systems infer tumour motion in the patient from observed external motion, stressing the need for assistance in this matter. Machine learning algorithms could be used to predict tumour motion in three-dimensional space. Corresponding models were analysed using CT data (Lin et al., 2019).

Entering the section on *positioning with SGRT systems*, the participants were asked about the information they needed at the linac to perform their task. Information about the immobilisation devices used (93.3 %), general patient data (84.4 %) and a profile picture of the patient on the couch (82.2 %) are desired as shown in Table A6 in the appendix 7.4. These attributes reach agreement values of more than 80 %. A static image of the patient with immobilisation devices (57.8 %) and superimposed reference position (53.3 %) seem to be complementary features reaching agreement values of slightly above 50 %. Also, the *display of the breathing curve* reaches an agreement value of 57.8 %. Additional information mentioned by the participants includes information on (1) necessary corrections of body regions, e.g., arm position, (2) automatic check of whether the correct immobilisation devices were used or correctly adjusted, and (3) the area of most profound inspiration. The participants rated acquisition, installation and integration costs as the main barriers to adopting SGRT systems, whereas a change in the treatment room design was not considered essential.

Camera-based SGRT systems pave the way for further innovations. Therefore, the participants were asked to predict the occurrence of selected new SGRT features. The results, as outlined in Table A7 in the appendix 7.4, reveal that the participants expect *patient recognition and verification* as future system advancements (80.0 %). The *real-time couch control* and *collision avoidance* features reach agreement values of 71.1 % and 62.2 %. However, the participants do not see a *fundamental change in immobilisation device usage* (31.1 %).

Finally, participants were asked to rate five statements according to their level of agreement. For better readability, the answers *I strongly agree* and *Agree* as well as *I disagree* and *I strongly disagree* have been combined into single values. The highest agreement values are reached for the statements *The demands on patient positioning are constantly increasing* (97.8 %) and *The accuracy of patient positioning is more important than the time required* (86.7 %). In contrast to this, the statements *A procedure without markers is important to patients* (37.8 %), *A non-technical room feeling in the treatment room is important to patients* (37.8 %) and *A procedure without radiation is important to patients* (31.1 %) reached only low agreement values. According to one of the medical physicists, most patients do not have enough knowledge to judge the technical and procedural differences. Patient education by system vendors and the trend towards more informed patients could change this in the future (Gutiontov et al., 2021; Vision RT, 2021a).

### 5.3. Empirical data – design aspects

In the following, the PACT concept was used to elaborate on requirements for developing interactive assistance systems for patient positioning. Users differ in their cognitive abilities, perceptions, and sensibilities. Therefore, we first evaluated the people dimension with the results shown in Table 10. The simplicity of *learning and operating the system* is a necessity and reaches an agreement value of 100 %. A system that is *intelligent and able to learn* is important for just 42.2 % of the participants. Similarly, an *appealing design* (42.2 %), *customisation features* (40.0 %) and *joy when using the system* (40.0 %) do not reach high agreement values with an equal number of participants having a neutral opinion on these features.

**Table 10.** Analysis of design requirements in the people dimension (*n* = 45).

Statement	Agree	Neutral	Not agree	Other
The system must be easy to learn and operate.	**100.0**	**0**	**0**	**0.0**
The system must be intelligent and able to learn.	42.2	40.0	15.6	2.2
The system must have an appealing design.	42.2	53.3	2.2	2.2
The system must be customisable.	40.0	35.6	22.2	2.2
The operation must be fun.	40.0	37.8	20.0	2.2

Characteristics of activities should be considered when creating interactive systems (Benyon, 2020). The importance of selected features were rated by the participants and is presented in Table 11 in descending order of agreement values. It is noteworthy that all participants want to have *decision-making power over the system*. The agreement ratings also confirm the core functions of modern SGRT systems, including *alerts to errors*, *high position accuracy*, *respiratory gating*, *intra-fraction beam on/off* and *documentation features*. All these features reach high agreement values of 86.7 to 97.8 %. High importance is also attributed to *cybersecurity* in particular (93.3 %). As many as 77.8 % consider the *support of stereotactic irradiation* as important or very important, which places higher demands on positioning accuracy. Also, 68.9 % of the participants state that the system *must reduce the positioning time*. Finally, 66.7 % agree that *the system should intervene in case of errors*.

**Table 11.** Analysis of design requirements in the activity dimension (*n* = 45).

Statement	Agree	Neutral	Not agree	Other
The user should have decision-making power over the system.	**100.0**	**0.0**	**0.0**	**0.0**
The system must alert to errors.	**97.8**	**2.2**	**0.0**	**0.0**
The system must increase the positioning accuracy.	**93.3**	**6.7**	**0.0**	**0.0**
The system must be protected against cyberattacks.	**93.3**	**6.7**	**0.0**	**0.0**
The system shall support respiratory gating.	**91.1**	**8.9**	**0.0**	**0.0**
The system must intervene in case of intra-fraction patient movement (beam on/off).	**86.7**	**13.3**	**0.0**	**0.0**
The system must document procedures.	**86.7**	**11.1**	**2.2**	**0.0**
The system must support stereotactic treatment.	**77.8**	**15.6**	**6.7**	**0.0**
The system must reduce the positioning time.	68.9	28.9	2.2	0.0
The system should intervene in case of errors.	66.7	24.4	8.9	0.0

Subsequently, the influencing factors given by the context of the activities were examined. These include physical, social and organisational aspects of radiotherapy as shown in Table 12. The *safety aspect* is with a 82.2 % agreement value most important to the participants. They also consider an *improved interaction of imaging and linac* as an important aspect. As many as 62.2 % of the respondents also agree that the *reputation of the radiotherapy centre is enhanced by SGRT systems*. However, they do not believe that SGRT deployment has a *positive effect on employee retention*. Concerning the *reduction of patients and employee waiting times*, the results show no clear preference.

**Table 12.** Analysis of design requirements in the context dimension (*n* = 45).

Statement	Agree	Neutral	Not agree	Other
The safety of the user is increased.	**82.2**	**13.3**	**4.4**	**0.0**
The interaction of imaging and linac is improved.	**71.1**	**20.0**	**6.7**	**2.2**
Reputation is enhanced by the system.	62.2	31.1	2.2	4.4
The waiting times of patients are reduced.	40.0	35.6	22.2	2.2
Employee retention is increased through the use of the system.	26.7	44.4	24.4	4.4
The waiting times of employees are reduced.	24.4	46.7	22.2	6.7

Finally, the respondents evaluated the technological aspects of patient positioning systems as presented in Table 13. According to the results, patient positioning systems must have *standardised interfaces for integration* purposes. This fact is consistent with the statement that systems must be *compatible with all linacs* (93.3 %). Most medical physicists would also prefer the *operation of the system to be location independent* (68.9 %) and the *QA to be as automatic as possible* (66.7 %). In contrast, the *control of the light in the treatment room* is not perceived as a mandatory feature.

**Table 13.** Analysis of design requirements in the technology dimension (*n* = 45).

Statement	Agree	Neutral	Not agree	Other
The system must have standardised interfaces for integration.	**100.0**	**0.0**	**0.0**	**0.0**
The system must be compatible with all linacs.	**93.3**	**6.7**	**0.0**	**0.0**
The operation of the system must be location-independent.	68.9	24.4	6.7	0.0
The quality assurance of the system must be as automatic as possible.	66.7	22.2	11.1	0.0
The system must be able to control the light in the room.	15.6	53.3	31.1	0.0

A specific aspect of the technology dimension is the user interface for the patient positioning system. The medical physicists were asked to indicate their preferred system interface for data input and information output to derive recommendations for new patient positioning assistance systems. The results are presented in Tables 14 and 15. By far, the participants prefer a *tablet PC* as the interface for system control (71.1 %). *Voice control* (17.8 %) and *virtual reality glasses* (15.6 %) are less desired alternatives for system control. The participants used the category *other* (31.1 %) with a free text field input to propose vendor-specific terminals, touch panels, remote control and standard PC with mouse and keyboard.

**Table 14.** Preferred interfaces for system control (*n* = 45, multiple selection).

Interface	Frequency	Percent
Tablet	32	**71.1**
Voice control	8	17.8
Virtual reality glasses	7	15.6
Gesture control	3	6.7
Mobile phone	1	2.2
Smart watch	0	0.0
Other	14	31.1

**Table 15.** Preferred media for system information (*n* = 45, multiple selection).

Interface	Frequency	Percent
Monitor in control room	41	**91.1**
Monitor at the wall	35	**77.8**
Projection onto the patient	30	66.7
Tablet	26	57.8
Virtual reality glasses	8	17.8
Haptic feedback	3	6.7
Voice output	2	4.4
Mobile phone	1	2.2
Smart watch	0	0.0
Other	1	2.2

Respondents show clear preferences when asked for their preferred interface for information presentation. A *monitor in the control room* is necessary for the participants (91.1 %). Slightly less, but still, 35 participants (77.8 %) would like to have a *monitor mounted on the wall* in the treatment room. *Direct projection onto the patient* is desired by 30 participants (66.7 %). The tablet solution, so preferred for data input, achieves lower approval ratings as an information output device (57.8 %). The solutions *virtual reality glasses*, *haptic feedback*, *voice output*, *mobile phone* and *smartwatch* reach only low agreement values. Furthermore, one participant mentioned a PC with a touch panel for the presentation of information.

The systematic literature review and the results of the structured online survey provide a solid basis for formulating our system design proposal.

## 6. Design of a patient positioning artefact

In this section, we formulate design requirements that form the basis of our design work and guide us in developing a subsequent artefact of a patient positioning system for radiotherapy practice. We present a conceptual model of the system and apply it to a specific use case, evaluating both the model and the use case in expert discussions and desktop research.

### 6.1. Design requirements

The systematic literature review and the structured online survey on patient positioning systems add wisdom to the knowledge base of our DSR project. Our conceptual patient positioning system proposes a new solution for the known problem of patient positioning in radiotherapy, thus representing an improvement according to Gregor and Hevner (2013). We propose design requirements supporting thereby the transition to the development stage. These design requirements are based on our analysis and integrate the agreement values of our empirical study leading to a distinction between MUST (acceptance above 65 percent), SHOULD (acceptance 50 to 64 percent) and COULD (acceptance below 50 percent) requirements. Additionally, we have created the following categories of design requirements: *general system requirements* (Table 16), *system integration requirements* (Table 17), *HMI requirements* (Table 18), *feature requirements* (Table 19) and *safety requirements* (Table 20).

**Table 16.** General design requirements for patient positioning systems.

Requirements	Description
DR 1.1	The system MUST increase patient positioning accuracy.
DR 1.2	The system MUST reduce patient positioning time.
DR 1.3	The system MUST prioritise patient positioning accuracy against patient positioning time.
DR 1.4	The user MUST have decision-making power over the system.
DR 1.5	The system SHOULD be designed to increase the reputation of the radiotherapy centre.
DR 1.6	The system COULD be designed to support a non technical room feeling.
DR 1.7	The system COULD have an appealing design.
DR 1.8	The system COULD be intelligent and able to learn.
DR 1.9	The system COULD reduce waiting times for employees and patients.
DR 1.10	The system COULD be marker less and not apply irradiation to patients.
DR 1.11	The system SHOULD be affordable to support system deployment worldwide.

**Table 17.** Design requirements for system integration.

Requirements	Description
DR 2.1	The system MUST support multiple linacs of different suppliers in private and public cancer centres worldwide.
DR 2.2	The system MUST have standardised interfaces for integration purposes.
DR 2.3	The system MUST improve the interaction of imaging modalities and linac.
DR 2.4	The system MUST support automated task execution such as quality assurance and procedure documentation.
DR 2.5	The system MUST be integrated to support real-time couch control.
DR 2.6	The system SHOULD support collision avoidance with the linac.

**Table 18.** Design requirements for human-machine interface.

Requirements	Description
DR 3.1	The system MUST be easy to learn and operate.
DR 3.2	The system MUST be able to be operated from a mobile device.
DR 3.3	The system MUST have a tablet computer for system control.
DR 3.4	Information MUST be displayed on a monitor in the control room.
DR 3.5	The system MUST embrace a wall mounted monitor in the treatment room.
DR 3.6	In-situ projection onto the patient body MUST be provided.
DR 3.7	The system SHOULD also present information on a tablet computer.
DR 3.9	The system MUST present a list of immobilisation devices used for the positioning of the patient at the linac.
DR 3.10	The system MUST present patient data including a profile picture.
DR 3.11	The system SHOULD present an image of the patient with immobilisation devices and superimposed reference position.
DR 3.12	The system SHOULD display the breathing curve of the patient.
DR 3.13	The system COULD be customisable and make the operation enjoyable.

**Table 19.** Design requirements for system features.

Requirements	Description
DR 4.1	The system MUST support respiratory gating techniques.
DR 4.2	The system MUST support stereotactic treatment.
DR 4.3	The system MUST detect movement of patients during treatment.
DR 4.4	The system MUST validate the correct usage of immobilisation devices.
DR 4.5	The system COULD predict tumour movement without marker inside the body.

**Table 20.** Design requirements for system safety.

Requirements	Description
DR 5.1	The system MUST increase user safety in patient positioning for radiotherapy practice.
DR 5.2	The system MUST recognise and validate patients to increase safety and avoid unnecessary work steps.
DR 5.3	The system MUST alert to errors.
DR 5.4	The system MUST intervene in case of errors.
DR 5.5	The system MUST stop the treatment in case of intra-fractional movement of the patient.
DR 5.6	The system COULD cover a large region of the patients body and recognise body changes.
DR 5.7	The system MUST be protected against cyberattacks.

### 6.2. Design artefact

**Figure 11.**
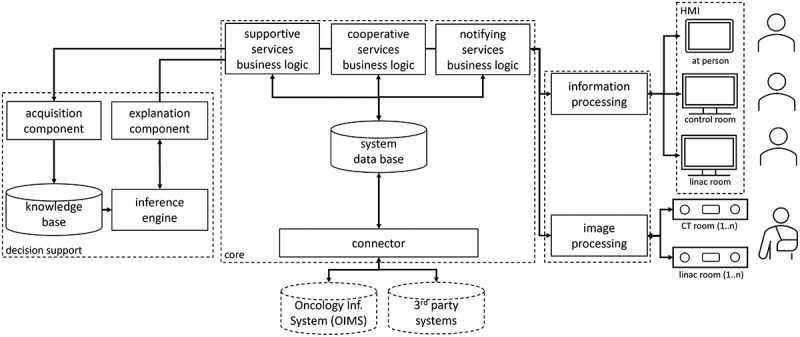
System overview of patient positioning artefact (own figure).

The empirical evidence gained in the SLR and online survey led to the formulation of design requirements forming the basis of developing the patient positioning artefact. Figure 11 shows the system architecture of our conceptual assistance system for patient positioning.

The architecture consists of four main parts: (1) The core consisting of three business logic units and the connectors, (2) information processing and image processing units, (3) the HMI, and (4) decision support units.

As part of the core, the notifying business logic secures time-critical alert services according to DR 5.3 - DR 5.5, including an emergency stop, the “linac beam off”.

The cooperative business logic implements services where the user interacts with the assistance system as described in DR 3.9 - DR 3.12. Physical activities, including interaction with the patient, often characterise those tasks. The activities can be presented in the form of previously defined user guidance, in which the system guides the user step-by-step through a work process.

The supportive services business logic implements user services that help the staff fulfil their administrative or configuration tasks. Examples are import and export functions and rules definition for tasks automation (DR 2.4). Both the supportive and cooperative services can be implemented as wizards or forms for data input and by using affordance properties (Maier & Fadel, 2009). The supportive and cooperative business logic can also provide expert advice in collaboration with the decision support unit, which includes a central knowledge base, a knowledge acquisition component, an inference engine and an explanation component. Thereby, it is possible to implement decision support services that assist the underlying business logic.

The decision support unit can utilise artificial intelligence according to DR 1.8 to support, among others, the use cases of *potential linac collisions with patients* (DR 2.6) and *predication of tumour motion during treatment* (DR 4.5). Artificial intelligence in radiotherapy has already been tested in previous work of our research group in the context of a COVID-19 chatbot for business continuity management (Reuter-Oppermann et al., 2021).

The information processing unit is connected to a user tablet for location-independent system operation and PC monitors in the control and linac room according to the DR 3.2 - DR 3.5 and DR 3.7. Two monitors could be well-positioned and wall mounted in the linac room to avoid non-ergonomic head movements. Stereotactic cameras with built-in projectors are installed in the CT and the linac rooms. Their network communication capacity and the connected image processing unit support a seamless and efficient workflow (DR 1.9). The projectors are used not only for sensory reasons (DR 1.1, DR 1.10, DR 4.1 - DR 4.4, DR 5.2, DR 5.5, DR 5.6) but also for augmenting information onto the patient body (DR 3.6), supporting system ergonomics for the users. The image processing unit uses the point cloud data of the cameras in the CT and linac room to calculate the body surface known from traditional SGRT systems. The body surface forms the basis for patient positioning and respiratory gating applications. Selected data and metadata are stored in the central system database and made available for processing by the three business logic units (a) notifying services, (b) cooperative services, and (c) supportive services.

Finally, the connector of the conceptual system for patient positioning secures the safe integration to the OIMS or other third-party systems via the standardised interfaces DICOM and HL7 according to DR 2.1 - DR 2.3, DR 2.5 and DR 5.7. The HMI of the patient positioning system should be designed to secure high usability addressing the DR 1.4, DR 1.5, DR 1.7, DR 3.1, and DR 3.13. The publications of Benyon (2020) on user experience design and Stephanidis et al. (2019) on the challenges of human-computer Interaction provide comprehensive input supporting future implementations based on the conceptual system.

### 6.3. Use cases

**Figure 12.**
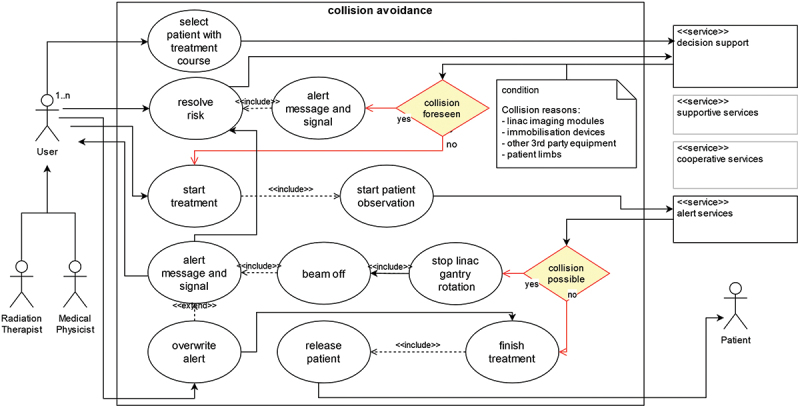
Collision avoidance use case of the patient positioning artefact (own figure).

Specific use cases of the conceptual system can be further detailed in the following instantiation work. As one example, Figure 12 visualises the specific use case of *collision avoidance* according to DR 2.6 in Unified Modeling Language (UML) (Object Management Group, 2017). The collision of a patient with moving parts of the linac, such as the gantry and build-on imaging devices, must be avoided. Various approaches have been investigated over the last decades. Using treatment techniques such as non-coplanar radiotherapy as described in Smyth et al. (2019) with changing angles of the patient couch increases the risk of patient-linac collisions. Reliable collision avoidance mechanisms should therefore be integrated into the clinical workflow. Modern TPS can simulate collision scenarios already during treatment planning based on geometric linac models and the patient body surface acquired at the CT scan (Islam et al., 2020; Miao et al., 2020; Wang et al., 2021). Additional research focuses on collision avoidance measures in the linac room to further increase patient safety (Cardan et al., 2017).

The use case of *collision avoidance* as shown in Figure 12 describes the interaction of the user, the system services and the patient under treatment for the application scenario. The supportive and cooperative services are shaded in grey. Related services can be utilised in real-life implementations to increase the user experience. For example, the user dialogue for *the selection of patient with treatment course* can be realised as a supportive wizard. Cooperative services can guide the user in the order of tasks necessary for *releasing the patient*. Functionally, as described in Figure 11, the decision support and alert services are embodied in this use case and highlighted in the evaluation. The decision support service warns the user before starting the treatment of a likely collision between the patient, the linac and other devices. The artificial intelligence-based service can utilise irradiation planning data, data about imaging and third-party equipment, data about pre-existing conditions of the patient, including pain and body characteristics, and general patient data, including demographics. Following a foreseen collision, the user can take action and resolve the risk. In case of no foreseen collision, the user starts the treatment. At this point, the alert service starts collision observation and intervenes in case of risk with a stop of the linac movement and patient irradiation. An overwrite function is incorporated if the user wants to proceed with the treatment while consciously accepting the risk. The use case thus demonstrates the possibilities of the interaction of the system services.

### 6.4. Design evaluation

The conceptual patient positioning system and the exemplary *collision avoidance* use case were evaluated in one-to-one expert discussions with three medical physicists, two of them being head physicists of radiotherapy departments of university hospitals in Germany. Each of the three medical physicists has more than 20 years of experience in radiotherapy. They are heading teams of 5–10 medical physicists and did not participate in the online survey. Both, the conceptual system and the exemplary use case were verified for correctness and reflected against the clinical experience. In the evaluation discussions, the necessity for the specific use case and the conceptual system’s flexibility and application possibilities were confirmed. However, the evaluation also extended the *collision avoidance* use case with the *overwrite alert* function. According to the medical physicists, this function is necessary in practice, as the user should always have control over the system under his or her own responsibility. A statement that confirms the correctness of DR 1.4 but was initially not considered in the use case design. In addition to the threefold structure of the business logic consisting of notifying, cooperative and supportive services, further research opportunities arising from the connection to the decision support unit were highlighted that can lead to other improvements of existing SGRT systems in the market (Hoisak et al., 2020). The interviewees mentioned the possibility of integrating the system with the imaging modalities of the linac, which is expected to become more important with the introduction of new treatment methods.

**Figure 13.**
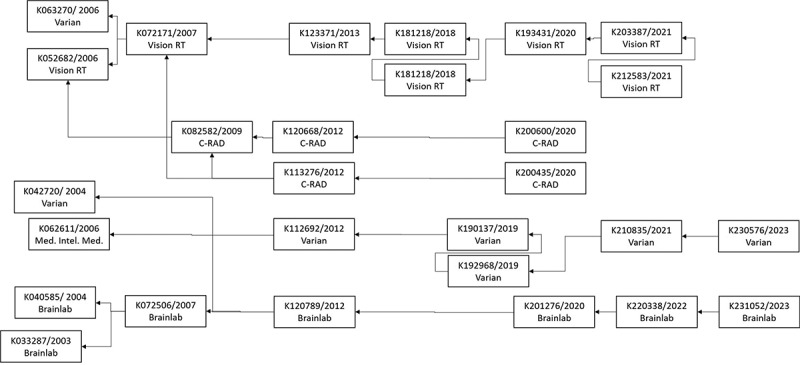
510(k) premarket notification dependencies of commercial SGRT solutions (own figure).

Furthermore, to evaluate the novelty of the artefact, we have conducted desktop research comparing the system architecture components as shown in Figure 11 with the commercial systems available on the market and with the systems identified in the SLR. We have orientated ourselves to the US for the commercially available systems, being home to around 25 percent of all linacs in operation worldwide and thereby being the single largest RT market worldwide (IAEA International Atomic Energy Agency, 2024). We analysed the 510 (k) premarket notification approval documents shown in Figure 13 of the FDA US Food and Drug Administration database FDA.2024, which are a prerequisite for marketing medical equipment in the US. We complemented our findings by analysing the internet pages of the medical device manufacturers.

The analysis of the systems from the manufacturer’s Vision RT, C-RAD, Varian and Brainlab shows that the systems are equipped with different sensors, such as x-ray, laser-scanners, IR-, thermal-, Cherenkov- and 3D stereo-vision cameras (Brainlab, 2022; C-RAD, 2022; Varian, 2021; Vision RT, 2021a). All systems employ various HMIs in the control and linac room. Some systems also allow location-independent control via tablet computer or include patient devices for specific breath-holding techniques. System connectors are described for importing general patient data and DICOM files from CT systems and TPS. Furthermore, some systems can correct the patient’s couch position or control the linac to support gated RT (linac beam on/off). All systems support alert and basic supportive services incorporated in the system software. More advanced support services are applied for breath-hold techniques. Only a few cooperative services are described, including patient validation, immobilisation device recognition or patient positioning support using augmented information on a real-time patient video. No decision support services in the form of the presented artefact are described so that its novelty is shown.

Following, we evaluated our artefact against the systems identified in the SLR. The evaluation highlights the variety of sensor technologies, including video pattern projection and photogrammetric cameras (Calow et al., 2002), structured light (Posada-Gomez et al., 2007), ToF cameras (Gilles et al., 2016) and IR cameras (Fuse et al., 2017), as well as vision markers and environmental scanning (Cosentino et al., 2017). Alert and support services for motion recognition are described by Calow et al. (2002), and cooperative services for patient positioning by Fuse et al. (2017). HMIs and connectors are only described by Cosentino et al. (2017), noting a general deficiency of detailed HMI and connector descriptions in most systems. Similar to the analysis of the commercial systems, none of the systems describes decision support services in the form of the presented artefact, proving its novelty.

Summarising, the design of our patient positioning artefact includes a new element of the decision support component incorporated in the SGRT conceptual model. It strengthens the knowledge base in the field of patient positioning in radiotherapy and healthcare in general. At the same time, innovation will further drive the development of SGRT systems. Developments in camera technology, plus the combination with other sensors, will lead to higher positioning accuracies. Incorporating the Cherenkov camera as described in the patent of Hale et al. (2020) is exemplary for this development. As users are confronted with an increasing number of complex systems in their daily workflow, significant improvements in human-computer interaction are also likely to be realised, resulting in more efficient and safe system usage.

## 7. Conclusion

The systematic literature review and online survey on camera-based SGRT systems provide a solid foundation for our design work. We formulated design requirements and created an information system artefact to improve the current state of radiotherapy practice and contributed to the academic knowledge base for patient positioning systems for cancer treatment.

### 7.1. Knowledge contribution

The work described in this paper answers the two formulated research questions. The systematic literature review results show the extent of academic publications about developing patient positioning systems employing application-oriented design science research. The review revealed only a few DSR articles focusing on the radiotherapy process step *therapy* and artefacts of the type *model*. However, the corresponding content analysis on the identified literature increased our design knowledge in the problem and solution space according Vom Brocke et al. (2020) and informed our subsequent empirical work. The online survey results and our design work outlined which design requirements should be considered to develop new assistance systems for patient positioning. The methods applied are rigorous and according to Leidner (2020) original in their composition.

The design requirements and system architecture model presented in this paper contribute to the *improvement area* according to Gregor and Hevner (2013) and support the development of new solutions in the application domain of radiotherapy. The knowledge abstracted in the form of prescriptive design requirements and architecture provides *level two* λ knowledge according to Drechsler and Hevner (2018); Gregor and Hevner (2013) and increases the solution design knowledge according to Drechsler and Hevner (2018). It could be further generalised as design principles in future academic research for the application in similar healthcare areas characterised by a high degree of technology, expert staff and risk profile.

### 7.2. Practical contribution

The developed system architecture helps to understand and compare existing SGRT systems. Furthermore, the proposed architecture with the decision support component allows the development of new applications for patient positioning in radiotherapy, representing improvements for staff and patients. The use case of *collision avoidance* was used to verify an exemplary use case that exposes patients to risk during treatment.

The studied phenomenon of patient positioning is essential for radiotherapy as an important form of cancer treatment. Thus, our work addresses a real-world problem that impacts society, considering that almost every six deaths in 2020 were caused by cancer (*Cancer: Key facts*, *2022*). The empirical data and results of the design work can be directly applied, which promises high usability by practitioners.

### 7.3. Discussion of limitations

Like all projects, this research has its limitations. First, the systematic literature review focused on information systems in radiotherapy, searching specifically for DSR artefacts. Despite a comprehensive search, it is possible that relevant literature was not discovered. Moreover, it cannot be ruled out that further relevant literature was published using alternative research frameworks. Second, we concentrated our survey on the German market. The survey could be conducted in other regions of the world with reasonable effort. The differences between high-, medium- and low-income countries will lead to variations in results because of the different technical and non-technical conditions. This fact applies particularly to SGRT systems that require additional investment, competencies and changes in existing work processes. Furthermore, the sample group is not representative and research participants might not reflect the entire opinion of the surveyed institution. Third, the exemplary evaluation of the *collision avoidance* use case did not consider potential *supportive and cooperative services* that can enhance the user experience. The complete system architecture, including these services and the remaining system components, was evaluated in desktop research. However, it cannot be ruled out that new SGRT developments have not yet been communicated to the market. In addition, the system architectures of commercial systems are not publicly available, meaning that the system architecture had to be inferred from the services. Fourth, we did not distinguish between different linac technologies and treatment regimes. Bore-type linacs have a considerably different design compared to C-arm linacs. The different construction methods put line-of-sight constraints on camera-based SGRT systems, which have not been considered in this conceptual work. Finally, treatment techniques with higher doses in fewer treatment fractions require high position accuracy. Hypofractionation and future flash therapy will generate new design requirements for patient positioning systems. The extension of the three-cycle model by the fourth cycle of *change and impact* by Drechsler and Hevner (2016) allows coping with dynamic application contexts and complex settings with many stakeholders, such as in radiotherapy. Extending the environmental analysis and distinguishing between *immediate application context* and *socio-technical system context* could help better analyse the impact of dynamic changes in technology and new radiotherapy treatment regimes being discussed in the medical physicist’s community.

### 7.4. Summary and outlook

Our research project aimed to understand the state of knowledge of patient positioning systems for radiotherapy practice and to identify design requirements to support the development of new systems. Little has been published in the DSR literature on patient positioning systems, although they are essential for efficient and safe radiotherapy. The publications mainly refer to therapy decision-making and planning. This is especially noticeable since DSR is a problem-solving paradigm focusing on real-life problems. Patient positioning includes many manual tasks in different locations in the radiotherapy centre. Camera-based SGRT systems in the imaging and treatment room provide important features and offer opportunities for further development. The analysis of the identified literature shows several technological approaches. However, the analysis revealed that little attention had been paid to the people and context dimensions in the developments. In contrast, there have been numerous studies on the dimensions of activities and technology. The PACT analysis reflected the existing knowledge and created new one. The elaborated design requirements guide the system design. The empirical evidence of the SLR and online survey served as a basis for developing the conceptual model of a positioning system supporting accurate and reproducible positioning of patients in the digitally networked radiotherapy environment. The proposed conceptual assistance system closes the gap between technology and the individual skills of specialists by applying varying degrees of intelligence and interactivity. The user services are implemented in the supportive, cooperative and notifying services. The core functionalities of positioning the patient on the linac are fulfilled, and the conceptual system supports collision avoidance and other advanced services. For the implementation, special attention should be paid to a high user experience.

The requirements for patient positioning are constantly increasing, strengthening the necessity for future research on assistance systems. The required reduction in process times must not be achieved at the expense of accuracy and treatment results, especially as requirements are expected to increase due to new treatment regimes. Positioning with SGRT systems takes longer compared to traditional laser systems. The intelligent combination of both could make use of the advantages of both technologies and resolve the contradiction between the benefits of SGRT technology on the one hand and the longer positioning time on the other hand. These aspects and their interplay could be investigated in additional research. Future work will strengthen the knowledge base with additional quantitative and qualitative findings. We will verify and potentially complement the design requirements and formulate design principles. A digital representation of the patient positioning system would create additional possibilities, such as optimising processes and resources using artificial intelligence. Thereby, patient positioning tasks in radiotherapy could become more efficient and safe. Finally, we plan to generalise our work further and formulate a practical conceptual framework for user assistance systems in work-intensive healthcare applications.

## Appendix

### Systematic literature review

In the following, the SLR-identified literature is presented.

Calow et al. (2002) described a photogrammetric system capturing patient surface data in real-time. The system allowed reproducible patient set-up and intra-fraction patient monitoring. It consisted of two cameras and one projector and was based on the photogrammetric evaluation of stereo-image pairs by projecting white light fringes onto the patient’s body. The raw data of the 3D measurement was a sequence of point clouds that could be evaluated in combination with other data modes common in radiotherapy for diagnosis and treatment planning. The system could be used as an additional verification tool for the correct patient positioning.

Posada et al. (2007) used 3D CT data and data points on the patient’s face for positioning. The data points were acquired with a structured light sensor in the treatment room. The coordinate systems of the CT and the 3D sensor data were linked to each other. A calibration of the sensor position gave the relationship between the coordinate systems. The position in the CT-coordinate system was sufficient to predict the tumour position in the linac coordinate system, with the applied algorithm being proven in terms of accuracy and reliability in several tests.

Posada-Gomez et al. (2007) presented a method for calibrating a 3D surface sensor independently of its measuring principle. They used the cloud of points directly to retrieve the extrinsic parameters, relating the virtual sensor space with the three laser beams of the radiotherapy room. A comparison between two calibration objects was presented. The use of 3D points supported flexible, accurate and robust extrinsic calibration.

Gilles et al. (2016) evaluated the patient positioning accuracy using a stereo-time of flight (ToF) camera system. The system used two cameras to scan the body surface to position patients on the treatment couch. The obtained point clouds were registered to detect intra-fraction motion and predict the displacement to place the patient in its reference position. The measures were compared to the effectively applied translations. The authors concluded that the proposed system provided sufficient accuracy and faster patient repositioning. Its capability to track the complete patient’s surface in real time allowed accurate positioning and real-time tracking of patient intra-fraction motion.

Fuse et al. (2017) developed an interactive patient position guidance and acquisition control system comprising infrared (IR) cameras, IR markers and dedicated software. IR markers were placed on the body surface, and their positions were calculated using vectors of three translational and three rotational parameters. The intra-fractional error for each marker was acquired with and without respiratory motion. The inclusion of multiple positioning markers allowed real-time visualisation of the patient’s posture, with feedback on misalignment and required postural adjustments.

Cosentino et al. (2017) developed an augmented reality tool to view the position of the patient on the couch in comparison to the computer-generated plan data. Due to the high availability and low cost of the hardware (iPad, Android tablet, MS HoloLens), the tool represents a promising solution to position patients in radiotherapy and to visualise medical information. After several test series, however, it was concluded that only rough positioning is possible due to measurement deviations.

### Structured questionnaire

In the following, the structured questionnaire with 21 questions is presented. The questionnaire contains the sections (1) general information, (2) positioning at linac, (3) positioning with SGRT, and (4) People-Activity-Context-Technology (PACT). The questionnaire’s opening, closing and chapter transitions are not shown for better readability.

**General information**

1. My radiotherapy centre is a…

- public hospital
- private hospital
- private radiotherapy centre
- ambulatory care centre

2. How many linear accelerators (linacs) do you operate in total?

3. How many radiotherapy therapists work in your radiotherapy centre? (full-time equivalents)

4. How many patients do you treat per year?

- Less than 400
- 401 to 700
- 701 to 1,000
- More than 1,000

**Positioning at the linac**

5. Which patient positioning systems do you currently use? How often do you use them? [Multiple selection possible, data in percent of the total number of patients]

- Laser
- Image-guided radiotherapy (CBCT, EPID, ultrasonic, kv/MV imaging)
- Surface-guided radiotherapy (camera, surface scanner)
- Other

6. Which patient positioning systems do you expect to use in two years from now? Approximately how often will you use them? [Multiple selection possible, data in percent of the total number of your patients]

- Laser
- Image-guided radiotherapy (CBCT, EPID, ultrasonic, kv/MV imaging)
- Surface-guided radiotherapy (camera, surface scanner)
- Other

7. What is the minimum accuracy that your positioning system must secure? [mm]

8. Please state the minimum motion of patients that a system must be able to detect. [mm]

9. Please state the minimum body surface area that an SGRT system should observe? [cm${\;}^{2}$]

10. How much time do you currently spend exclusively for positioning a patient at the linac? [min.]

11. What measures or systems would simplify or improve the positioning of patients at the linac?

**Positioning with SGRT**

12. What information is important to you when working at the linac? [multiple selection possible]

- Patient data
- Profile picture of the patient
- List of immobilisation devices used
- Image of the patient with superimposed reference position
- Static image of the patient with immobilisation devices
- Display of the breathing curve
- Other

13. Please rank the following factors in order of importance as barriers to SGRT system adoption. [where 1 = most important]

- Cost for installation and integration
- Additional effort for the introduction of the system
- Management does not see a need
- Changed appearance of the linac room
- System acquisition cost
- Additional effort for the application

14. In the future, SGRT systems will… [multiple selection]

- be able to recognise and verify patients.
- use position deviations for real-time couch control.
- fundamentally change the use of immobilisation devices.
- support collision avoidance with the linac.

15. Please rate the following statements. [I strongly agree, agree, neutral, disagree, strongly disagree, don’t know]

- The accuracy of patient positioning is more important than the time required.
- A procedure without markers is important to patients.
- A procedure without radiation is important to patients.
- A non-technical room feeling in the treatment room is important to patients.
- The demands on patient positioning are constantly increasing

### PACT - People

16. How important are the following aspects of a patient positioning assistance system to you? Please rate the following statements… [I strongly agree, agree, neutral, disagree, strongly disagree, don’t know]

- The system must be easy to learn and operate.
- The operation must be fun.
- The system must be personalizable.
- The system must be intelligent and able to learn.
- The system must have an appealing design.

### PACT - Activities

17. How important are the following aspects of a patient positioning assistance system to you? [I strongly agree, agree, neutral, disagree, strongly disagree, don’t know]

- The system must increase the positioning accuracy.
- The system must reduce the positioning time.
- The system must alert to errors.
- The system should intervene in case of errors.
- The system must support stereotactic treatment.
- The system shall support respiratory gating.
- The system must intervene in case of intra-fraction patient movement (beam on/off).
- The user should have decision-making power over the system.
- The system must document procedures.
- The system must be protected against cyberattacks.

### PACT - Context

18. How important are the following aspects of a patient positioning assistance system to you? [I strongly agree, agree, neutral, disagree, strongly disagree, don’t know]

- Reputation is enhanced by the system.
- Employee retention is increased through the use of the system.
- The safety of the user is increased.
- The waiting times of patients are reduced.
- The waiting times of employees are reduced.
- The interaction of imaging and linac is improved.

### PACT - Technology

19. How important are the following aspects of a patient positioning assistance system to you? [I strongly agree, agree, neutral, disagree, strongly disagree, don’t know]

- The system must be compatible with all linacs.
- The operation of the system must be location-independent.
- The system must be able to control the light in the treatment room.
- The system must have standardised interfaces for integration.
- The quality assurance of the system must be as automatic as possible.

20. Which user interfaces do you prefer to control the system? [multiple selection]

- Tablet
- Mobile phone
- Smart watch
- Virtual reality glasses
- Voice control
- Gesture control
- Other

21. Which technology should be used to display the information of the system? [multiple selection]

- Tablet
- Mobile phone
- Smart watch
- Virtual reality glasses
- Monitor at the wall
- Monitor in control room
- Projection onto the patient
- Voice output
- Haptic feedback
- Other

End of the questionnaire.

### Empirical data

In the following, additional empirical data from the online survey is presented.

Table A1 presents the structure of the sample group with a balanced distribution of private and public institutions.

**Table A1.** Structure of the sample group (*n* = 45).

Type of RT centre	Frequency	Percent
Public hospital	18	40.0
Private hospital	6	13.3
Private radiotherapy centre	10	22.2
Ambulatory radiotherapy centre	10	22.2
Others	1	2.2

The number of patients treated per year is depicted in Table A2, showing that 66.7 % of the sample group treats more than 1,000 patients annually. Only 13.3 % of the participants treat up to 700 patients per year. The radiotherapy centres in the sample group operate on average 2.5 linacs, with a maximum of seven linacs in one of the large hospitals. On average, 5.3 radiotherapy therapists support the operations of one linac.

**Table A2.** Number of patients treated per year (*n* = 45).

Number of patients	Frequency	Percent
> 1,000	30	**66.7**
701–1,000	9	20.0
401–700	5	11.1
<400	1	2.2

Table A3 presents the usage of positioning systems in the years 2020 and 2022.

**Table A3.** Usage of positioning systems 2020 vs. 2022 (*n* = 45, multiple selection).

Systems 2020	Frequency	Percent
IGRT (CBCT, EPID, ultrasonic, kv/MV imaging)	45	100.0
Laser systems	41	91.1
SGRT (camera, surface scanner)	16	35.6
Others	6	13.3
**Systems 2022**		
IGRT (CBCT, EPID, ultrasonic, kv/MV imaging)	45	100.0
Laser systems	34	75.6
SGRT (camera, surface scanner)	32	71.1
Others	8	17.8

Table A4 presents the expected change in the frequency of usage of patient positioning systems within the two-year time frame.

**Table A4.** Frequency of positioning system usage 2020 vs. 2022 (*n* = 45).

Systems 2020	Frequency	Percent	Standard deviation
IGRT	41	81.5	27.1
Laser	34	87.2	29.0
SGRT	14	54.4	34.9
**Systems 2022**			
IGRT	35	78.2	28.5
Laser	27	77.4	35.1
SGRT	22	66.5	30.8

Table A5 presents the times needed for patient positioning depending on the system used.

**Table A5.** Time required for positioning task (*n* = 42).

System	Frequency	Minutes	Standard deviation
All systems	42	5.3	3.1
Only laser	28	4.3	2.0
Laser and SGRT	12	6.1	3.8
Only SGRT	3	9.0	5.4

Table A6 presents the required information at the linac.

**Table A6.** Required information at the linac (*n* = 45, multiple selection).

Attribute	Frequency	Percent
List of immobilisation devices used	42	**93.3**
Patient data	38	**84.4**
Profile picture of the patient	37	**82.2**
Static image of the patient with immobilisation devices	26	57.8
Display of the breathing curve	26	57.8
Image of the patient with superimposed reference position	24	53.3
Other	3	6.7

Table A7 presents potential new features of SGRT systems.

**Table A7.** Potential new features of SGRT systems (*n* = 45, multiple selection).

Feature	Frequency	Percent
Recognise and verify patients	36	**80.0**
Use position deviations for real-time couch control	32	71.1
Support collision avoidance with the linac	28	62.2
Fundamental change of immobilisation device usage	14	31.1

## References

[cit0001] American Association of Physicists in Medicine. (2021, October 03). *AAPM committee tree - task group No. 302 - surface image guided radiotherapy*. Retrieved October 3, 2021, from https://www.aapm.org

[cit0002] Anderson, G., Doherty, R., & Ganapathy, S. (2011). *Design, user experience, and usability: Theory, methods, tools and practice*. Springer.

[cit0003] ATLAS.ti. (2021, May 15). *Atlas.Ti: The qualitative data analysis and research software*. Retrieved May 15, 2021, from https://atlasti.com/

[cit0004] Atun, R., Jaffray, D. A., Barton, M. B., Bray, F., Baumann, M., Vikram, B., Hanna, T. P., Knaul, F. M., Lievens, Y., Lui, T. Y. M., Milosevic, M., O’Sullivan, B., Rodin, D. L., Rosenblatt, E., Van Dyk, J., Yap, M. L., Zubizarreta, E., & Gospodarowicz, M. (2015, September). Expanding global access to radiotherapy. *The Lancet Oncology*, 16(10), 1153–1186. 10.1016/S1470-2045(15)00222-3 26419354

[cit0005] Batista, V., Meyer, J., Kügele, M., & Al-Hallaq, H. (2020). Clinical paradigms and challenges in surface guided radiation therapy: Where do we go from here? *Radiotherapy and Oncology: Journal of the European Society for Therapeutic Radiology and Oncology*, 153, 34–42. 10.1016/j.radonc.2020.09.041 32987044

[cit0006] Benyon, D. (2020). *Designing user experience: A guide to hci, ux and interaction design* (4th ed). Pearson.

[cit0007] Berufenet medizinphysiker/in. (2022, October 03). Retrieved October 3, 2022, from https://web.arbeitsagentur.de/berufenet/beruf/59541

[cit0008] Brainlab. (2022, January 20). *Exactrac dynamic: A new dimension in patient tracking and monitoring*. Retrieved July 27, 2021, from https://www.brainlab.com/radiosurgery-products/exactrac/

[cit0009] Calow, R., Gademann, G., Krell, G., Mecke, R., Michaelis, B., Riefenstahl, N., & Walke, M. (2002). Photogrammetric measurement of patients in radiotherapy. *Isprs Journal of Photogrammetry & Remote Sensing*, 56(5–6), 347–359. 10.1016/S0924-2716(02)00070-9

[cit0010] *Cancer: Key facts*. (2022, November 07). Retrieved November 12, 2022, from https://www.who.int/news-room/fact-sheets/detail/cancer

[cit0011] Cardan, R. A., Popple, R. A., & Fiveash, J. (2017). A priori patient-specific collision avoidance in radiotherapy using consumer grade depth cameras. *Medical Physics*, 44(7), 3430–3436. 10.1002/mp.12313 28474757

[cit0012] CITAVI. (2021, April 17). *Citavi - reference management and knowledge organization*. Retrieved April 17, 2021, from https://www.citavi.com/en

[cit0013] Clarke, R., Davison, R. M., & Jia, W. (2020). Researcher perspective in the is discipline: An empirical study of articles in the basket of 8 journals. *Information Technology & People*, 33(6), 1515–1541. 10.1108/ITP-04-2019-0189

[cit0014] Cooper, H. M. (1988). Organizing knowledge syntheses: A taxonomy of literature reviews. *Knowledge in Society*, 1(1), 104–126. 10.1007/BF03177550

[cit0015] Cosentino, F., John, N. W., & Vaarkamp, J. (2017). Rad-ar: Radiotherapy - augmented reality: An augmented reality tool for radiotherapy implemented on consumer electronics devices. In *Proceedings - 2017 International Conference on Cyberworlds, CW 2017 - in cooperation with: Eurographics Association International Federation for Information Processing ACM SIGGRAPH*, *2017-January*.

[cit0016] C-RAD. (2022, January 20). *C-Rad advanced radiation therapy — C-Rad*. Retrieved July 27, 2021, from https://c-rad.se/

[cit0017] Cram, W. A., Templier, M., & Pare, G. (2020). (re)considering the concept of literature review reproducibility. *Journal of the Association for Information Systems*, 21(5), 1103–1114. https://aisel.aisnet.org/jais/vol21/iss5/10

[cit0018] DEGRO. (2021, May 23). *Deutsche Gesellschaft für Radioonkologie e.V*. Retrieved May 23, 2021, from https://www.degro.org/

[cit0019] Drechsler, A., & Hevner, A. (2016). A four-cycle model of is design science research: Capturing the dynamic nature of is artifact design, 1–8.

[cit0020] Drechsler, A., & Hevner, A. R. (2018). Utilizing, producing, and contributing design knowledge in DSR projects. In Designing for a Digital and Globalized World: 13th International Conference, DESRIST 2018, Chennai, India, June 3–6, 2018, Proceedings 13 (pp. 82-97). Springer International Publishing.

[cit0021] Dresch, A., Lacerda, D. P., & Antunes Ju´nior, J. A. (2015). *Design science research: A method for science and technology advancement*. Springer International Publishing. Retrieved May 15, 2020, from https://link.springer.com/content/pdf/10.1007%2F978-3-319-07374-3.pdf

[cit0022] Ferlay, J., Colombet, M., Soerjomataram, I., Mathers, C., Parkin, D. M., Piñeros, M., Znaor, A., & Bray, F. (2019). Estimating the global cancer incidence and mortality in 2018: Globocan sources and methods. *International Journal of Cancer*, 144(8), 1941–1953. 10.1002/ijc.31937 30350310

[cit0023] Field, M., Hardcastle, N., Jameson, M., Aherne, N., & Holloway, L. (2021). Machine learning applications in radiation oncology. *Physics and Imaging in Radiation Oncology*, 19, 13–24. 10.1016/j.phro.2021.05.007 34307915 PMC8295850

[cit0024] Fuse, H., Komatsu, K., Arakawa, H., Sakae, T., & Tatsuya, F. (2017). An infrared interactive patient position guidance and acquisition control system for use during radiotherapy treatment. *Journal of Radiotherapy in Practice*, 16(3), 303–310. 10.1017/S1460396917000140

[cit0025] Gastaldi, L., Corso, M., Gastaldi, L., & Corso, M. (2012). Smart healthcare digitaliza- tion: Using ICT to effectively balance exploration and exploitation within hospitals. *International Journal of Engineering Business Management*, 4, 9. https://cyberleninka.org/article/n/1507476

[cit0026] Gilles, M., Fayad, H., Miglierini, P., Clement, J. F., Scheib, S., Cozzi, L., Bert, J., Boussion, N., Schick, U., Pradier, O., & Visvikis, D. (2016). Patient positioning in radiotherapy based on surface imaging using time of flight cameras. *Medical Physics*, 43(8), 4833–4843. 10.1118/1.4959536 27487901

[cit0027] Gläser, J., & Laudel, G. (2012). *Experteninterviews und qualitative inhaltsanalyse als instrumente rekonstruierender untersuchungen* (4th ed.). Springer VS. http://d-nb.info/1002141753/04

[cit0028] Gregor, S., & Hevner, A. R. (2013). Positioning and presenting design science research for maximum impact. *MIS Quarterly*, 37(2), 337–355. 10.25300/MISQ/2013/37.2.01

[cit0029] *Guidelines for the certification of clinically qualified medical physicists*. (2021). International Atomic Energy Agency. https://www.iaea.org/publications/14746/guidelines-for-the-certification-of-clinically-qualified-medical-physicists.

[cit0030] Guo, W., Muller-Polyzou, R., Chen, Z., Meier, N., & Georgiadis, A. (2020). Patient positioning in radiotherapy. In *Memea 2020 Conference Proceedings* (pp. 1–6). IEEE, Piscataway, NJ, USA.

[cit0031] Gutiontov, S. I., Golden, D. W., McCloskey, S., Shumway, D., Sullivan, D. R., Wall, T. J., Gunderson, L. L, & Jagsi, R. (2021). Informed consent in radiation oncology. *International Journal of Radiation Oncology, Biology, Physics*, 109(1), 29–35. https://www.redjournal.org/article/S0360-3016(20)34223-1/fulltext 32911020 10.1016/j.ijrobp.2020.08.064

[cit0032] Hale, G. M., Meir, I. D., Pawlicki, T., & Allen, M. (2020). *Radiation dosage monitoring system* (Nos. US 10,675,485 B2).

[cit0033] Hevner, A., & Chatterjee, S. (2010). Design research in information systems: Theory and practice (vol. 22). Springer US. http://site.ebrary.com/lib/alltitles/docDetail.action?docID=10396983.

[cit0034] Hevner, A. R. (2007). A three cycle view of design science research. *Scandinavian Journal of Information Systems*, 19(2), 87–92.

[cit0035] Hevner, A. R., March, S. T., Park, J., & Ram, S. (2004). Design science in information systems research. *MIS Quarterly*, 28(1), 75–105. 10.2307/25148625

[cit0036] Hoang Thuan, N., Drechsler, A., & Antunes, P. (2019). Construction of design science research questions. *Communications of the Association for Information Systems*, 44(1), 332–363. 10.17705/1CAIS.04420

[cit0037] Hoisak, J. D. P., & Pawlicki, T. (2018). The role of optical surface imaging systems in radiation therapy. *Seminars in Radiation Oncology*, 28(3), 185–193. 10.1016/j.semradonc.2018.02.003 29933878

[cit0038] Hoisak, J. D. P., Paxton, A. B., Waghorn, B., & Pawlicki, T. (2020). *Surface guided radiation therapy*. CRC Press.

[cit0039] Hombrink, G., & Promberger, C. (2020). *How and why surface guided radiation therapy developed*. Retrieved February 10, 2021, from https://www.brainlab.com/de/journal/how-and-why-surface-guided-radiation-therapy-developed-sgrt/

[cit0040] Hsieh, H.-F., & Shannon, S. E. (2005). Three approaches to qualitative content analysis. *Qualitative Health Research*, 15(9), 1277–1288. 10.1177/1049732305276687 16204405

[cit0041] IAEA International Atomic Energy Agency. (2024, January 25). *Dirac directory of radiotherapy cen- tres*. Retrieved February 3, 2024, from https://dirac.iaea.org/Query/Countries

[cit0042] *Iec 62366-1:2015: Medical devices — part 1: Application of usability engineering to medical devices*. (2015). Geneva. Retrieved February 4, 2022, from https://www.iso.org/standard/63179.html

[cit0043] The International Agency for Research on Cancer. (2024, February 03). *Global cancer observatory*. Retrieved November 13, 2022, from https://gco.iarc.fr/

[cit0044] Islam, N., Kilian-Meneghin, J., deBoer, S., & Podgorsak, M. (2020). A collision prediction framework for noncoplanar radiotherapy planning and delivery. *Journal of Applied Clinical Medical Physics*, 21(8), 92–106. 10.1002/acm2.12920 32559004 PMC7484832

[cit0045] *Iso 14971:2019: Medical devices — application of risk management to medical devices*. (2019). Geneva. Retrieved February 4, 2022, from https://www.iso.org/standard/72704.html

[cit0046] Leidner, D. E. (2020). What’s in a contribution? *1536–9323* , 238–245.

[cit0047] Liberati, A., Altman, D. G., Tetzlaff, J., Mulrow, C., Gøtzsche, P. C., Ioannidis, J. P. A., Clarke, M., Devereaux, P. J., KleijnenMike, J., & Moher, D. (2009). The prisma statement for reporting systematic reviews and meta- analyses of studies that evaluate health care interventions: Explanation and elaboration. *PLOS Medicine*, 62(10), e1–e34. 10.1016/j.jclinepi.2009.06.006 19631507

[cit0048] Limesurvey. (2021, April 17). *Limesurvey - easy online survey tool*. Retrieved April 17, 2021, from https://www.limesurvey.org/

[cit0049] Lin, H., Zou, W., Li, T., Feigenberg, S. J., Teo, B.-K.-K., & Dong, L. (2019). A super-learner Model for tumor motion prediction and management in radiation therapy: Development and feasibility evaluation. *Scientific Reports*, 9(1), 14868. 10.1038/s41598-019-51338-y 31619736 PMC6795883

[cit0050] Maedche, A., Morana, S., Schacht, S., Werth, D., & Krumeich, J. (2016). Advanced user assistance systems. *Business & Information Systems Engineering*, 58(5), 367–370. 10.1007/s12599-016-0444-2

[cit0051] Maier, J. R. A., & Fadel, G. M. (2009). Affordance based design: A relational theory for design. *Research in Engineering Design*, 20(1), 13–27. https://link.springer.com/article/10.1007/s00163-008-0060-3

[cit0052] March, S. T., & Smith, G. F. (1995). Design and natural science research on information technology. *Decision Support Systems*, 15(4), 251–266. 10.1016/0167-9236(94)00041-2

[cit0053] Mayring, P. (2000). *Qualitative inhaltsanalyse: Grundlagen und techniken* (7th ed., Vol. 1). Deutscher Studien Verlag.

[cit0054] Miao, J., Niu, C., Liu, Z., Tian, Y., & Dai, J. (2020). A practical method for predicting patient-specific collision in radiotherapy. *Journal of Applied Clinical Medical Physics*, 21(8), 65–72. 10.1002/acm2.12915 PMC7484822 32462733

[cit0055] Moher, D., Liberati, A., Tetzlaff, J., & Altman, D. G. (2009). Preferred reporting items for systematic reviews and meta-analyses: The prisma statement. *PLOS Medicine*, 6(7), e1000097. 10.1371/journal.pmed.1000097 19621072 PMC2707599

[cit0056] Morana, S., Dehlin, T., Reuter-Oppermann, M., & Sunyaev, A. (2017). *User As- sistance for health care information systems*. https://www.researchgate.net/publication/321136891.

[cit0057] *Mpe in der strahlen- und brachytherapie*. (2022, October 03). Retrieved October 3, 2022, from https://www.jmp.dgmp.de/preview/de-DE/1032/strahlenbrachytherapie/

[cit0058] Müller-Polyzou, R., Reuter-Oppermann, M., Engbert, A., & Schmidt, R. (2021). Identifying user assistance systems for radiotherapy to increase efficiency and help saving lives. *Health Systems*, 10(4), 318–336. 34745592 10.1080/20476965.2020.1803148 PMC8567950

[cit0059] Murphy, J. (2018). Nursing and technology: A love/hate relationship. *Nursing Economic$*, 28(6), 405–408. 21291063

[cit0060] Nations, U. (2021, April 17). *World population prospects - population division*. Retrieved April 17, 2021, from https://population.un.org/wpp/

[cit0061] Object Management Group. (2017, December). *Unified modeling language, V2.5.1: Version 2.5.1* (*No. formal/2017-12-05*). www.omg.org/spec/UML/

[cit0062] Ostern, N., Perscheid, G., Reelitz, C., & Moormann, J. (2021). Keeping pace with the healthcare transformation: A literature review and research agenda for a new decade of health information systems research. *Electronic Markets*, 31(4), 901–921. 10.1007/s12525-021-00484-1 35599689 PMC8285287

[cit0063] Padilla, L., Havnen-Smith, A., Cervin˜o, L., & Al-Hallaq, H. A. (2019). A survey of surface imaging use in radiation oncology in the United States. *Journal of Applied Clinical Medical Physics*, 20(12), 70–77. 10.1002/acm2.12762 PMC6909172 31743588

[cit0064] Parkinson, C., Matthams, C., Foley, K., & Spezi, E. (2021). Artificial intelligence in radiation oncology: A review of its current status and potential application for the radiotherapy workforce. *Radiography*, 27(1), S63–S68. https://www.sciencedirect.com/science/article/pii/S1078817421000924 34493445 10.1016/j.radi.2021.07.012

[cit0065] Posada, R., Daul, C., Wolf, D., Aletti, P., & Townsend, D. (2007). Towards a noninvasive intracranial tumor irradiation using 3d optical imaging and multimodal data registration. *International Journal of Biomedical Imaging*, 2007(1). 10.1155/2007/62030 PMC2267930 18364992

[cit0066] Posada-Gomez, R., Alor-Hernandez, G., Garcia-Martinez, M., & Quintana-Silva, J. (2007). An extrinsic calibration method for 3d range surface sensors: An application in radiotherapy patient positioning. In *17th International Conference on Electronics, Communications and Computers* (pp. 18–21). IEEE.

[cit0067] Reuter-Oppermann, M., Müller-Polyzou, R., & Georgiadis, A. (2021). Towards a decision support system for radiotherapy business continuity in a pandemic crisis. *Journal of Decision Systems*, 31(1–2), 40–67. 10.1080/12460125.2021.1947946

[cit0068] Richter, A., Sweeney, R., Baier, K., Flentje, M., & Guckenberger, M. (2009). Effect of breathing motion in radiotherapy of breast cancer: 4d dose calculation and motion tracking via epid. *Strahlentherapie und Onkologie: Organ der Deutschen Rontgengesellschaft*, 185(7), 425–430. 10.1007/s00066-009-1980-1 19714303

[cit0069] Rosenblatt, E., & Zubizarreta, E., & Eds. (2017). *Radiotherapy in cancer care: Facing the global challenge*. IAEA. https://ebookcentral.proquest.com/lib/gbv/detail.action?docID=4921048.

[cit0070] Schlegel, W., Karger, C. P., & J¨akel, O. (2018). *Medizinische physik*. Springer Berlin Heidelberg.

[cit0071] Schnell, R., Hill, P. B., & Esser, E. (2013). *Methoden der empirischen sozialforschung* (10th ed.). Oldenbourg Verlag.

[cit0072] SGRT Community. (2020, February 28). *Sgrt and safert automation in radiotherapy*. Retrieved January 23, 2022, from https://www.youtube.com/watch?v=ZbVMqcONRFg

[cit0073] Skripcak, T., Belka, C., Bosch, W., Brink, C., Brunner, T., Budach, V. … Baumann, M. (2014). Creating a data exchange strategy for radiotherapy research: Towards federated databases and anonymised public datasets. *Radiotherapy and Oncology: Journal of the Eu- Ropean Society for Therapeutic Radiology and Oncology*, 113(3), 303–309. https://www.sciencedirect.com/science/article/pii/S0167814014004071 10.1016/j.radonc.2014.10.001 PMC4648243 25458128

[cit0074] Smyth, G., Evans, P. M., Bamber, J. C., & Bedford, J. L. (2019). Recent developments in non-coplanar radiotherapy. *The British Journal of Radiology*, 92(1097), 20180908. https://pubmed.ncbi.nlm.nih.gov/30694086/30694086 10.1259/bjr.20180908 PMC6580906

[cit0075] Stephanidis, C., Salvendy, G., Antona, M., Chen, J. Y. C., Dong, J., Duffy, V. G. … Zhou, J. (2019). Seven hci grand challenges. *International Journal of Human–Computer Interaction*, 35(14), 1229–1269. 10.1080/10447318.2019.1619259

[cit0076] Tranfield, D., Denyer, D., & Smart, P. (2003). Towards a methodology for developing evidence- informed management knowledge by means of systematic review. *British Journal of Management*, 14(3), 207–222. 10.1111/1467-8551.00375

[cit0077] Vaishnavi, V. K., & Kuechler, W. (2015). *Design science research methods and patterns: Innovating information and communication technology* (2nd ed). CRC Press Taylor & Francis Group.

[cit0078] van Aken, J., Chandrasekaran, A., & Halman, J. (2016). Conducting and publishing design science research. *Journal of Operations Management*, 47–48(1), 1–8. 10.1016/j.jom.2016.06.004

[cit0079] Varian. (2021, July 27). *Identify*. Retrieved July 27, 2021, from https://www.varian.com/products/radiotherapy/real-time-tracking-motion-management/identify

[cit0080] Varian. (2022, January 15). *Halcyon*. Retrieved January 15, 2022, from https://www.varian.com/products/radiotherapy/treatment-delivery/halcyon

[cit0081] Vision RT. (2021a, August 05). *Alignrt education*. Retrieved August 5, 2021, from https://www.saferradiationtherapy.com/

[cit0082] Vision RT. (2021b, July 27). *Sgrt community*. Retrieved July 27, 2021, from https://sgrt.org/

[cit0083] Vision RT. (2021c, May 27). *Vision rt - innovative solutions to improve radiation therapy*. Retrieved July 27, 2021, from https://www.visionrt.com/

[cit0084] Vom Brocke, J., Winter, R., Hevner, A., & Maedche, A. (2020). Special issue editorial–accumulation and evolution of design knowledge in design science research: A journey through time and space. *Journal of the Association for Information Systems*, 21(3), 520–544. 10.17705/1jais.00611

[cit0085] Wang, Y.-J., Yao, J.-S., Lai, F., & Cheng, J. C.-H. (2021). Ct-based collision prediction software for external-beam radiation therapy. *Frontiers in Oncology*, 11, 617007. 10.3389/fonc.2021.617007 33777756 PMC7991715

[cit0086] Webster, J., & Watson, R. T. (2002). Analyzing the past to prepare for the future: Writing a literature review. *MIS Quarterly*, 26(2), 13–23.

[cit0087] Wooster Community Hospital. (2021). *Does radiation oncology have fewer side effects compared to chemotherapy? — Wooster Community Hospital*. Retrieved November 5, 2022, from https://www.woosterhospital.org/does-radiation-oncology-have-fewer-side-effects-compared-to-chemotherapy/

[cit0088] World Health Organization, & International Atomic Energy Agency (Eds.). (2020, February 03). Who report on cancer: Setting priorities, investing wisely and providing care for all. Retrieved January 22, 2022, from https://www.who.int/publications/i/item/who-report-on-cancer-setting-priorities-investing-wisely-and-providing-care-for-all

